# Age‐related behavioral and molecular landmarks in new mouse models for studying Alzheimer's disease in Down syndrome

**DOI:** 10.1002/alz.71498

**Published:** 2026-05-21

**Authors:** Monika Rataj Baniowska, Paige Mumford, Francesca Prestia, Pauline Stephan, Millie Beament, Marie‐Christine Birling, Chiara Lanzillotta, Letizia Ciafardini, Eugenio Barone, Gloria Lau, Claire Chevalier, Chadia Nahy, Nadia Messaddeq, Thais Lestra, Yixing Wu, Valérie Nalesso, Fabio Di Domenico, Frances Wiseman, Yann Herault

**Affiliations:** ^1^ Institut de Génétique Biologie Moléculaire et Cellulaire, IGBMC, UMR 7104‐ UMR‐S 1258 Université de Strasbourg, CNRS, Inserm Illkirch France; ^2^ UK Dementia Research Institute at UCL London UK; ^3^ Department of Biochemical Sciences A. Rossi Fanelli Sapienza University of Rome Rome Italy; ^4^ Université de Strasbourg, CNRS, Inserm, PHEN‐Institut Clinique de la Souris, PHEN‐ICS, CELPHEDIA, UAR2062, US66 Illkirch France; ^5^ CNRS, CELPHEDIA Core, UAR2052 Villejuif France

**Keywords:** ageing, anxiety, brain metabolism, cognition, Down syndrome, learning and memory, mouse model, neurodegeneration

## Abstract

**INTRODUCTION:**

Down syndrome (DS) is the leading genetic cause of intellectual disability and Alzheimer's disease (AD), with over 90% of individuals developing AD‐related dementia (DSAD). The triplication of the *APP* gene on chromosome 21 drives early amyloid‐β (Aβ) accumulation, but other Hsa21 genes also contribute to pathology. Current DSAD models are limited by species‐specific Aβ differences.

**METHODS:**

We developed and characterized two novel DSAD mouse models with partial humanization of Aβ.

**RESULTS:**

These models exhibit early AD features: cognitive deficits, hyperactivity, altered novelty and risk responses, tau hyperphosphorylation, and endolysosomal dysfunction. Amyloid precursor protein (APP) processing shifts toward β‐secretase, increasing CTF‐β and altering Aβ dynamics. Aβ humanization modulates behavior, improving specific cognitive tasks but enhancing anxiety traits. Myelinosome formation and impaired autophagic flux further align these models with human AD pathology.

**DISCUSSION:**

They offer valuable tools to investigate early DSAD mechanisms and therapeutic strategies, pending development of a fully humanized trisomic model.

## Highlights

- Novel Down syndrome–Alzheimer's disease (DSAD) mouse models with humanized amyloid‐β (Aβ): Two new DSAD mouse models (Ts68Yah and Dp(16)15Yah) were developed, incorporating partial humanization of the Aβ peptide to better mimic human AD pathology.
- Behavioral modulation by Aβ humanization: Aβ humanization improves performance in specific cognitive tasks but exacerbates anxiety‐related traits, revealing complex genotype–phenotype interactions relevant to DSAD progression.
- Recapitulation of early AD features: These models exhibit hallmark features of early AD, including behavior changes with hyperactivity, altered anxiety and risk‐taking behavior, Tau hyperphosphorylation, and endolysosomal dysfunction.
- Amyloid precursor protein (APP) processing shift and Aβ dynamics: Humanization of Aβ shifts APP processing toward the β‐secretase pathway, increasing CTF‐β levels and modulating Aβ abundance and aggregation across brain regions and age.
- Autophagic and myelinosome pathology: Ts68Yah mice show impaired autophagic flux and myelinosome formation, aligning with neurodegenerative processes observed in human AD and DS brains.

## 1 BACKGROUND

Down syndrome (DS) is the most common cause of intellectual disabilities, occurring in 1:700‐1:1000 births as a result of the trisomy of human chromosome 21 (Hsa21). The condition causes profound alterations to neurodevelopment, affecting the function of multiple brain cell types throughout life.^1^ This results in deficits in various cognitive domains, alterations in brain structure, delays in developmental milestones, and an increased risk of disorders of the brain, including epilepsy and autism.^2^, ^3^, ^4^ People who have DS also have an elevated risk of developing additional comorbidities.^5^, ^6^, ^7^, ^8^ In addition, the vast majority of individuals with DS will develop Alzheimer's disease (AD), with a mean age of clinical onset of 55 years; such that DS is now considered the most commonly occurring genetic cause of AD (DSAD).^9^, ^10^, ^11^, ^12^, ^13^, ^14^ DSAD neuropathology is first characterized by the accumulation of intraneuronal Aβ aggregates, followed by extracellular Aβ plaques, and then tau neurofibrillary tangles within the brain, which precede the early onset of dementia. Triplication of the amyloid precursor protein (*APP)* gene,^15^ which encodes the amyloid precursor protein, is a major driver of early Aβ accumulation in DS, a condition associated with AD. Conversely, the increased abundance of the APP cleavage product, Aβ, has been attributed to being a key factor in the early onset of disease.^16^ Changes in neuronal endosomal biology are an early and key component of DSAD pathogenesis and arise from the combined effects of *APP* triplication together with additional Hsa21 genes which regulate vesicular trafficking, endocytosis, and endosomal dynamics.^17^, ^18^, ^19^ However, the additional copy of other Hsa21 genes has been linked to alterations in AD‐relevant processes in preclinical models. The dual‐specificity tyrosine phosphorylation‐regulated kinase 1A (*DYRK1A*), an Hsa21 gene overexpressed in the brains of persons who have DS, targets many proteins involved in AD development and progression, including Tau phosphorylation.^20^, ^21^, ^22^ The Hsa21‐encoded β‐site APP‐cleaving enzyme 2, BACE2, is proposed to influence the production and/or clearance of Aβ and modulate Aβ accumulation in an organoid model of DSAD.^23^ However, recent studies in DS mouse models showed a limited influence of *Bace2* dosage.^24^, ^25^ Moreover, differences in neuropathology, gene expression, protein abundance, clinical presentation, and disease progression occur in DSAD compared with other forms of AD in the euploid population.^9^, ^10^, ^11^, ^12^, ^13^, ^14^ These differences result from the modulation of biology caused by the additional copy of genes encoded on Hsa21. A better understanding of the biology of DSAD is critical to inform safe AD therapy, as illustrated by usage guidance for anti‐Aβ monoclonal therapies, which precluded their use in people with DS because of safety concerns.^26^

Mouse models of DS have been key for investigating the cellular and molecular mechanisms underpinning DS‐associated intellectual disability^27^, ^28^, ^29^ in children and young adults. However, ageing is known to interact with the effect of DS‐associated additional gene content, resulting in age‐dependent disruptions of redox balance, stress responses, endosomal function, metabolic pathways, programmed cell death, and synaptic plasticity.^30^, ^31^, ^32^, ^33^, ^34^, ^35^ This may, in part, relate to the ageing‐mediated breakdown of protein homeostasis, causing enhanced proteome disruption in older individuals, and/or accelerated ageing mediated by trisomy 21.^36^, ^37^

In current DS mouse models, three copies of *App* recapitulate key aspects of DSAD, including elevation of APP and Aβ levels, perturbation of endolysosomal biology, ageing‐related loss of basal forebrain cholinergic neurons, and associated changes to glial cell marker expression.^38^, ^39^, ^40^ Nevertheless, these models have limitations, partly due to genetic differences in the Aβ sequence between rodents and humans, which prevent the recapitulation of some key aspects of AD neuropathology, such as the formation and deposition of Aβ and tau pathological aggregates, as well as associated cellular responses.^41^ Recent studies in AD rodent modeling have demonstrated that humanization of the Aβ sequence in euploid mouse and rat models leads to neuropathological and cognitive changes.^41^, ^42^, ^43^ Similar models are not yet available for DSAD. Here, we develop and characterize two novel DSAD mouse models to provide a greatly needed resource for preclinical DSAD research.

## 2 METHODS

### 2.1 Ethics approval

All animal research were done following the Directive of the European Parliament: 2010/63/EU, revising/replacing Directive 86/609/EEC and with French Law (Decree n° 2013‐118 01 and its supporting annexes entered into legislation February 01, 2013) relative to the protection of animals used in scientific experimentation at the “Institute Clinique de la Souris” an approved user institution under the agreement D67‐218‐40 valid until 14/10/2028 and the recommendation of the ARRIVES Guidelines 2.0.^44^ The Ministry of National Education, Higher Education and Research evaluated the ethical compliance of the project with our ethical committee n°17 and accredited the animal research with the two authorization APAFIS #15187‐201805221519333v5 and #15169‐2018052111497498v3.

### 2.2 Animals

Y.H.’s lab previously described Ts66Yah mice (MGI:6459295) in the B6C3BF1 genetic background.^29^ The model is available at Jax (RRID: IMSR JAX:036600). The second model used is the B6.129S7‐Dp(16Lipi‐Zbtb21)1Yey/J, named here Dp(16)1Yey (MGI:3714529), originally obtained from Dr. Eugene Yu,^28^ and maintained on the C57BL/6J genetic background. The humanized Aβ mouse model, called here *App^H^*, was generated by introducing three nucleotide modifications (GGA > CGA, TTT > TAT, and CGC > CAC) and inducing the respective amino‐acid changes G676R, F681Y, and R684H in the C57BL/6N ES cells to get the *App^H^* allele (Figure S1). Selected chimeras derived from homologous recombinant clones were bred with the C57BL/6N line to establish the *App^H^* line. The B6N *App^H^* was then backcrossed with C57BL/6J for two generations and intercrossed up to 10 generations. Then, the Ts66Yah was crossed twice to obtain B6C3BF1 [Ts66Yah, *App^H/H/+}^*], which we named Ts68Yah. These mice had three copies of *App*; two were humanized, and an additional wild‐type (WT) copy was present originally in the Ts66Yah minichromosome. Further crosses were attempted to mediate recombination of the humanized *App* allele with the mouse *App* carried by the Ts66Yah minichromosome. The Ts68Yah line was then maintained as *App^H/H^* homozygous by intercross (Figure S1). Here, *App^H/H^* homozygotes are called *App^H2^*. Similarly, Dp(16)1Yey mice were crossed with *App^H/H^* B6J/N N2F10 animals and then maintained on this genetic background. Here, Dp(16)1Yey with two humanized *App* alleles (*App^H/H/+^*) are named Dp(16)15Yah (Fiureg S1). All lines were therefore bred on sighted backgrounds to avoid blindness‐related confounding factors.

Animals were housed in groups of up to four males and five females per cage (cage type: Green Line, 39 × 20 × 16 cm, Tecniplast, Italy) and had free access to purified water and food (D04 chow diet, Safe D04, Augy, *France*). The temperature in the animal house was maintained at 23°C ± 1°C, and the light cycle was controlled as 12 hours (h) light and 12 h dark (lights on at 7 AM). For experimental groups with WT and mutant genotypes, no enrichment was added. Specific cohorts were produced to investigate phenotypes starting at 3 months (3 M), and a second one for 9 months with both sexes included (9 M). All the genotypes were analyzed at the same time (Table S1). Independent cohorts of mice were planned for histological studies at 3, 6, 9, and 20 months of age (Table S1). However, during our attempt to age mutant mice, we encountered welfare concerns, with several trisomic animals reaching ethical endpoints that required euthanasia. As a result, only a limited number of individuals (*n* = 3) were maintained for more detailed investigations at 20 Months.

**RESEARCH IN CONTEXT**

1. **Systematic review**: Down syndrome (DS) is the most common genetic cause of Alzheimer's disease (AD), yet existing DSAD mouse models fail to fully replicate human pathology due to species‐specific differences in amyloid‐β (Aβ) sequence. Previous models lacked extracellular Aβ deposition and tau pathology, limiting their translational relevance.
2. **Interpretation**: This study introduces two novel DSAD mouse models with partial humanization of Aβ, which exhibit key features of early AD: cognitive impairment, hyperactivity, tau hyperphosphorylation, and endolysosomal dysfunction. Humanized Aβ alters APP processing and modulates behavior, improving spatial memory while increasing anxiety‐related traits. These models better reflect the molecular and behavioral complexity of DSAD.
3. **Future directions**: Further development of fully humanized trisomic models is needed to capture the full spectrum of DSAD pathology. These models will be instrumental for testing targeted therapies and understanding genotype–phenotype interactions in AD progression within the context of DS.

### 2.3 Behavior analysis

The behavioral testing pipeline was identical to that previously described for the Ts66Yah model,^30^ and was conducted at the Mouse Clinical Institute by the same experimenter. Briefly, it included the procedure for circadian activity test, open field, elevated plus maze (EPM), Y‐maze, and new object recognition (NOR) with 1 h and 24 h of retention time, and the Morris water maze (MWM) test. On experimental days, animals were transferred to the experimental room 30 min before the start of the test. Except for circadian activity, all experiments were performed between 8 AM and 4 PM. Mice were given a resting time of 2–7 days between two different types of tests. The Ts66Yah behavioral analysis was undertaken using identical protocols at the same facility to reduce the number of animals used in research (as per 3R principles). Here, we present WT and Ts66Yah data that have been previously published, alongside previously unpublished data for the *App^H2^* and Ts68Yah lines. This is for the purpose of maximizing understanding of the effect of Aβ humanization in the DS models.

### 2.4 Tissue preparation for immunohistochemistry and electron microscopy of mouse hippocampi

Mice were anaesthetized and perfused by intracardial injection with cold phosphate buffer saline (PBS, 5 mL) followed by about 50 mL of 4% paraformaldehyde (PFA) in PBS. Each brain was dissected and post‐fixed with 4% PFA in PBSx1 at 4°C with gentle rocking overnight. After two rinses in cold PBS, one hemisphere was isolated for immunohistochemical analysis and the other for electron microscopy. We randomized the samples, but for each type of analysis, we took the same number of left/right hemispheres per genotype and per sex.

For immunohistochemistry analyses, either cryo‐protected frozen or paraffin‐embedded samples were used. For cryosection, hemispheres were put in 20% sucrose for 24 h at 4°C or until the hemisphere reached the bottom of the tube. Tissue was then rinsed with about 1 mL of distilled water and dried, prior to embedding in OCT compound (Tissue‐Tek OCT compound, ref. 4583) in HistoMold (13 × 19 mm; Leica) and freezing. Samples were then stored at ‐80°C until cryostat sectioning at 14 µm. Alternatively, tissue was paraffin‐embedded and stored at room‐temperature, prior to microtome sectioning at 5 µm. Sections were stained with Nissl or mouse monoclonal antibodies against APP: the anti‐APP N‐term 22C11 (Thermo Fisher Scientific, ref. 14‐9749‐80; RRID: AB_2572977; dilution 1/1000), 6E10 reactive to aa 1‐16 Aβ and to APP (with the epitope EFRHDS; Biolegend, ref. 803001; RRID: AB_2564653; dilution 1/1‐2000), 82E1 Amyloid β N‐terminal specific (DEMEDITEC Diagnostics GmbH; ref. 10323; dilution 1/500). Signals were detected with VECTASTAIN Elite ABC Rabbit IgG kit (Thermo Fisher Scientific, Illkirch, ref. NC9293436) under a light microscope (Leitz). Pictures were taken with a digital camera and processed with the Adobe Photoshop CS6 software.

For electron microscopy analysis, 250 µm‐thick sagittal sections were cut with a Vibratome, using a lateral to the middle of the brain cutting direction. Four to five sections were collected at the level of the hippocampus. Whole hippocampus was then dissected from the section, and tissue punches of the section were taken for the cortical regions of interest. Each piece of tissue was then post‐fixed in 1 mL of 4% PFA for 24 h, prior to storage in PBS at 4°C. No glutaraldehyde was used in the protocol to prevent the aggregation of endosomal structures.

### 2.5 Biochemical fractionation of mouse brain tissues for the quantification of Aβ by MSD immunoassay

Cortical proteins were fractionated as described in Shankar et al.^45^ A half cortex was weighed on a microscale and homogenized in three volumes of ice‐cold Tris‐buffered saline (TBS) (50 mM Tris‐HCl, pH 8.0, Thermo Scientific Chemicals, ref. J60877.K3) containing complete protease inhibitors (Roche, ref. 4693116001) and PhosSTOP phosphatase inhibitors (Roche, ref. 4906845001) using the handheld rotor‐stator homogenizer TissueRuptor II and disposable probes (Qiagen). 50 µL of homogenate was removed, snap‐frozen, and stored at −70°C for later use in Western blotting. The remaining homogenate was then transferred to 1.5 mL microfuge tubes (Beckman Coulter, ref. 357448), balanced by adding more TBS, and centrifuged at 186,000 × *g* with a Beckman Coulter Optima Max‐XP Benchtop ultracentrifuge fitted with fixed‐angle rotor TLA‐55 at 4°C for 30 min. Supernatant (the Tris‐soluble fraction) was removed and stored at ‐70°C. The remaining pellet was homogenized in three volumes, minus 50 µL, of ice‐cold 1% Triton X‐100 (Invitrogen, ref. HFH10) in TBS (50 mM Tris‐HCl, pH 8.0) using a handheld mechanical homogenizer and disposable pestles (Anachem), balanced and centrifuged at 186,000 × *g* for 30 min at 4°C. The resultant supernatant (the 1% Triton X‐100 soluble fraction) was removed and stored at ‐70°C. The pellet was then re‐suspended in three volumes (by original cortical weight), minus 50 uL, of TBS (50 mM Tris‐HCl pH8.0) containing 5 M guanidine HCl (Sigma Aldrich, ref. G3272‐25G) using a handheld mechanical homogeniser and disposable pestles, and left overnight at 4°C on a rocker to ensure full re‐suspension (the 5 M Gnd‐HCl soluble fraction), and subsequently stored at ‐70°C.

Aβ_40,_ and Aβ_42_ levels were quantified on Multi‐Spot 96‐well plates, pre‐coated with anti‐Aβ_38_, Aβ_40_, and Aβ_42_ antibodies, using a 6E10 detection antibody, using multiplex MSD technology (V‐PLEX Aβ Peptide Panel 1 (6E10) Kit, ref. K15200E‐1), as described in Wiseman et al.^46^ Quantities of analytes (pg/mL) were normalized to the original starting weight of cortical material per volume of buffer (mg/mL), resulting in final quantities of analytes per wet weight of cortical material (pg/mg) for statistical analysis as recommended in the original protocol and used by a number of groups studying AD in mouse models of amyloid accumulation.^42^, ^47^, ^48^, ^49^, ^50^

### 2.6 Western blot procedures

Total cortical and hippocampal protein was prepared in 1× RIPA buffer (Merck, ref. 20‐188), pH7.4, containing Tris‐HCl (50 mM pH = 7.4), NaCl (150 mM), 10% NP‐40, 2.5% deoxycholic acid, 10 mM ethylenediaminetetraacetic acid (EDTA), supplemented with cOmplete protease inhibitors (Roche, ref. 4693116001) and PhosSTOP phosphatase inhibitors (Roche, ref. 4906845001). The 50 µL of cortex TBS homogenate, generated before biochemical fractionation as described previously, was added to an equal volume of 2× RIPA buffer, while a whole hippocampus was homogenized in 1× RIPA buffer. Samples were mechanically homogenized on ice and then centrifuged at 21,300 × *g* for 30 min at 4°C to remove cellular debris. Using the Bradford method, the supernatant was then used to determine the total protein concentration (Bio‐Rad, ref. 5000006). Samples from individual animals were analyzed separately and were not pooled.

To analyze APP and APP C‐Terminal fragment (APP‐CTF) abundance, total brain proteins were then denatured in lithium dodecyl sulfate (LDS) denaturing buffer (Invitrogen, ref. NP0007) and β‐mercaptoethanol, prior to separation by sodium dodecyl sulfate‐polyacrylamide gel electrophoresis (SDS‐PAGE) gel electrophoresis using precast NuPAGE 4‐12% Bis‐Tris gels (Invitrogen, ref. NP0321BOX). Proteins were transferred to Trans‐Blot Turbo Mini 0.2 µm nitrocellulose membranes (Bio‐Rad, ref. 1704158) and then blocked in Intercept (PBS) Blocking Buffer (LI‐COR, ref. 927‐70001). Primary antibodies against APP (Sigma Aldrich, ref. A8717) and β‐actin (Sigma Aldrich, ref. A5441) were diluted in Intercept (PBS) Blocking Buffer. Membranes were incubated in primary overnight at 4°C. Secondary antibodies, goat anti‐mouse IRDye 680RD or goat anti‐rabbit IRDye 800CW (LI‐COR), were diluted 1:10,000 in Intercept (PBS) Blocking Buffer, and membranes were incubated in secondary solution for 1 h at room temperature. Blots were subsequently imaged by Odyssey CLx Imager (LI‐COR). Band density was analyzed using ImageJ. The relative signal of the antibody of interest compared to the internal loading control was then calculated, and this relative signal was normalized to the mean relative signal of the WT samples electrophoresed on the same gel. Means of technical replicates were calculated and used for analysis of variance (ANOVA), such that biological replicates were used as the experimental unit.

To analysis Tau, triplicated proteins and degradation pathways, 15 µg of proteins from mice hippocampi and cortices were resolved on Criterion TGX Stain‐Free 4%‐15%, 18‐well (Bio‐Rad, ref. 5678084) and 26‐well gel (Bio‐Rad, ref. 5678085) in a Criterion large format electrophoresis cell (Bio‐Rad, ref. 1656001) in Tris/Glycine/SDS (TGS) Running Buffer (Bio‐Rad, ref. 1610772). Immediately after electrophoresis, the gel was transferred to a Chemi/UV/Stain‐Free tray and then placed into a ChemiDoc MP Imaging System (Bio‐Rad, ref. 17001402). The gel was UV‐activated using Image Lab Software (Bio‐Rad) to collect the total protein load image. Following electrophoresis and gel imaging, the proteins were transferred via the TransBlot Turbo semi‐dry blotting apparatus (Bio‐Rad, ref. 1704150) onto nitrocellulose membranes (Bio‐Rad, ref. 162‐0115). The membranes were blocked with 3% bovine serum albumin (BSA) in 0.5% Tween‐20/TBS (TTBS) and incubated overnight at 4°C with the antibodies reported in Table S2. After 3 washes with TTBS buffer, the membranes were incubated for 1 h at room temperature with anti‐rabbit/mouse immunoglobulin G (IgG) secondary antibody conjugated with horseradish peroxidase (1:10,000; Sigma–Aldrich). Signals were detected with Clarity enhanced chemiluminescence (ECL) substrate (Bio‐Rad, ref. 1705061), then acquired with Chemi‐Doc MP, and band density was analyzed using Image Lab software (Bio‐Rad), taking advantage of the Bio‐Rad Stain‐free technology that allows for measuring total lane density as a loading control.

### 2.7 Immunofluorescence and image analysis

Half hemispheres of brain were fixed by immersion in 10% buffered formal saline, prior to cryopreservation in 30% sucrose/PBS (Sigma‐Aldrich) for minimum 72 h at 4°C, then embedded in CellPath Optimal Cutting Temperature (OCT) embedding matrix by immersion in 2‐methylbutane on dry ice. Two 30 µm sections per animal, were permeabilized in 1% Triton X‐100/PBS for 20 min, and 0.3% Triton X‐100/PBS for 30 min. Sections were blocked for two hours in 20% donkey serum/1% BSA/0.3% Triton X‐100/PBS, before incubation with anti‐human amyloid (N) (82E1) mouse IgG monoclonal antibody (IBL‐America, cat. 10379) at 1:500 dilution (final concentration 0.2 ug/mL) overnight at RT with agitation. After washing, slides were incubated with donkey anti‐mouse IgG (H+L) highly cross‐adsorbed secondary antibody‐Alexa Fluor 568 (Thermo Fisher Scientific, cat. A10037), at 1:500 dilution (final concentration 4 ug/mL) for 4 h at RT with agitation. Slides were then washed before incubation with 1:10,000 DAPI (cat. D1306) in 0.3% Triton X‐100/PBS for 5 min. Slides were washed and mounted with ProLong Gold Antifade Mountant (cat. P36930).

Slides were imaged with the ZEISS Axio Scan.Z1 with Z‐stack thickness of 31 µm at increments of 1 µm, using the Alexa Flour 568 and DAPI channels with automated focus and consistent imaging parameters across all animals. Following imaging, Zeiss Zen 3.6 software was used to pre‐process the images. The hippocampus and cortex of each section were manually segmented using a macro provided by ZEISS, creating a  .czi file of only hippocampus and only cortex for each tissue section, containing Z‐stacks of all channels. Each Z‐stack was compressed to a single plane using maximum intensity orthogonal projection and then separated into individual AF568 and DAPI TIFF images by split scene processing. On 16‐bit images of Aβ plaque staining, the percentage area positive for staining was determined using an ImageJ macro. A threshold of pixel value, representing pixels that were positive for Aβ deposition staining, was determined using a *App^NL‐G‐F^* positive control slide, with this threshold applied to all images to prevent biased calculation. A binary mask was generated, with all pixels passing the threshold assigned a value of 1 and all pixels below the threshold assigned a value of 0, to identify areas positive for staining. The number of pixels which passed the threshold out of the total number of pixels of the region of interest area were quantified as a percentage.

### 2.8 Statistical analysis

Behavioral data are presented as mean group value ± standard error of the mean (SEM). Normal distribution was evaluated with the Shapiro‐Wilk test. Differences were considered significant at *p* < 0.05. As no differences were found between males and females with ANOVA, the data were pooled. We performed an unpaired two‐tailed t‐test between genotypes or a repeated measures ANOVA (genotype, age) with the Bonferroni post hoc test using GraphPad Prism software (version 10.0). One‐sample t‐test was used in analysis of: (i) NOR test to evaluate recognition index against chance level (50%), (ii) MWM task to compare performance against chance level (25%), (iii) Y‐maze to evaluate spontaneous alternation behavior against chance level (50%). We identified the differentiating variables (Table S3) for the multivariate analysis, allowing us to separate the four genotypes using the GDAPHEN pipeline.^30^, ^51^ Here, we analyzed 12 selected behavioral variables, plus sex and genotype, derived from the mouse behavioral dataset with the groups of the same individuals analyzed at 3 and 9 months of age.

Molecular data were analyzed as indicated in figure legends by ANOVA with between‐subject factors being genetic status (WT/Ts68Yah or WT/Dp(16)15Yah, *App^+/+^* or *App^H2^*, and sex (female or male), or genetic status (WT vs. Ts66Yah, *App^H2^* vs. Ts68Yah, Ts66Yah vs. Ts68Yah, and WT vs. *App^H2^*). All timepoints were analyzed separately. Molecular data for Western blot quantification were calculated as a percentage of age‐matched WT, and are expressed as mean ± SEM. The subject means of technical replicates (Western blot or MSD assay well) were calculated and used in the ANOVA, such that independent mice were treated as the experimental unit. Bonferroni correction for multiple comparisons was used for all post hoc pairwise comparisons. Alpha was set at 0.05 and reported in the figure legends for the effects of genetic status. Columns were used to show differences among the groups (WT vs. *App^H2^*, *App^H2^* vs. Ts68Yah, Ts66Yah vs. Ts68Yah, WT vs. Ts66Yah). Dots were used for WT, squares for *App^H2^*, triangles for Ts66Yah, and rhombi for Ts68Yah. Red color indicates female mice and light blue color indicates male mice. Lines were used to show age‐associated changes within each group. For columns: *p < 0.05, **p < 0.01, ***p < 0.001, and ****p < 0.0001 (2‐way ANOVA with Bonferroni multiple comparisons). Statistical analyses and graphs were undertaken using IBM SPSS Statistics (version 26) and/or GraphPad Prism (version 10.0) software for univariate analysis. To evaluate the effects of genotypes, sex, and their interactions, within each age group, we performed a three‐way ANOVA analysis.

## 3 RESULTS

### 3.1 Humanization of three copies of the mouse Aβ sequence results in reduced life span in DS mouse models

The endogenous mouse Aβ sequence of APP is less able to aggregate compared with the human peptide, and does not result in the deposition of Aβ aggregates in the brain of mouse models, even in the presence of increased *APP* gene dose.^38^, ^39^ This limits the range of AD‐relevant phenotypes that can be studied in current mouse models of DS. To address this, we humanized the Aβ peptide sequence in two DS mouse models by modifying three codons in exon 14 using ES cell technologies to transform G676R, F681Y, and R684H, generating the recombinant Aβ allele, *App^H^* (Figure S1). We used conventional mouse breeding to introduce the mutation *App^H^* into the Ts66Yah mice line over two generations. The generated mice carried two copies of *App^H^* and the Ts66Yah minichromosome, resulting in the novel line Ts68Yah, equivalent to Ts66Yah with *App^H2^* (*App^+/H/H^*) allele. We attempted to generate a line carrying the Ts66Yah minichromosome with three copies of *App^H^* using in vivo recombination. We generated 1,045 offspring from Ts66Yah; *App^+/H/H^* parent crossed with *App^H/H^* parent. Of these, 403 offspring carried the Ts66Yah minichromosome, reflecting the expected transmission frequency of 38.5%.^29^ We identified five offspring carrying the WT unmodified *App^+/+^* allele, and two offspring that carried three copies of the *App^H^* allele (*App^H/H/H^*) that also carried the minichromosome. Accordingly, the observed frequency 1.2% (five out of 403) demonstrated that meiotic crossing over between the minichromosome and the genetically altered mouse chromosome 16 occurred at the expected recombination rate for this chromosomal region. However, these two recombinant‐minichromosome *App^H/H/H^* animals were females and died before successfully transmitting the Ts66Yah‐*App^H^* recombined minichromosome.

We undertook a similar strategy with the Dp(16)1Yey DS mouse model,^28^ using conventional breeding to generate offspring with a combined Dp(16)1Yey; *App^+/H/H^* genotype. We attempted in vivo recombination to generate offspring with a Dp(16)1Yey; *App^H/H/H^* genotype. We produced 698 animals from Dp(16)1Yey; *App^+/H/H^* crossed with *App^H/H^*, of which 504 were of the *App^H/H^* genotype and 194 of the Dp(16)1Yey; *App^+/H/H^* genotype, here called the Dp(16)15Yah. Thus, transmission of the segmental duplication was as expected (27.8%). We identified nine animals with a recombination of the *App* allele, four were WT with two or no humanized *App* alleles, and three were Dp(16)1Yey; *App^H/H/H^*. Unfortunately, all these Dp(16)1Yey; *App^H/H/H^* animals also died prematurely and did not generate any offspring. Thus, it was not possible to successfully age or breed DSAD models with three copies of humanized *App*.

Here we present a side‐by‐side comparison of the novel DSAD mouse model Ts68Yah compared with *App^H/H^* control animals, noted here after as *App^H2^*, and validate key findings in the Dp(16)15Yah model. These novel DSAD models, Ts68Yah and Dp(16)15Yah, carry respectively an additional copy of ∼ 103 and 117 of Hsa21 mouse protein‐coding gene orthologues and two copies of *App* expressing humanized Aβ. We used in vivo data collected at the same time but published previously from WT and Ts66Yah animals to minimize animal usage,^30^ to distinguish between the effects of Aβ humanization and the minichromosome on phenotype in the novel Ts68Yah model.

### 3.2 Humanizing two copies of Aβ peptide alters behavior in the Ts66Yah DS mouse model at 3 and 9 months of age

To understand the effect of Aβ humanization on DS and AD relevant behavioral phenotypes in the Ts68Yah mouse model of DSAD, we compared behavior in these animals to *App^H2^*, and previously generated results in the WT and Ts66Yah animals. Mice were analyzed for changes in circadian activity (CA), activity in a novel environment in the open field (OF), anxiety‐like behavior in the EPM, working memory using the Y‐maze test of spontaneous alternation, object recognition memory using the the NOR task and spatial memory using the MWM. Behavioral phenotyping was conducted on 165 mice of both sexes, divided into two independent cohorts tested at 3 and 9 months of age (WT: *n* = 22 males, *n* = 16 females; *App^H2^*: *n* = 22 males, *n* = 22 females; Ts66Yah: *n* = 21 males, *n* = 20 females; Ts68Yah: *n* = 21 males, *n* = 21 females). Two‐way ANOVA (Genotype and Sex as factors) revealed no significant effect of Sex.

**FIGURE 1:**
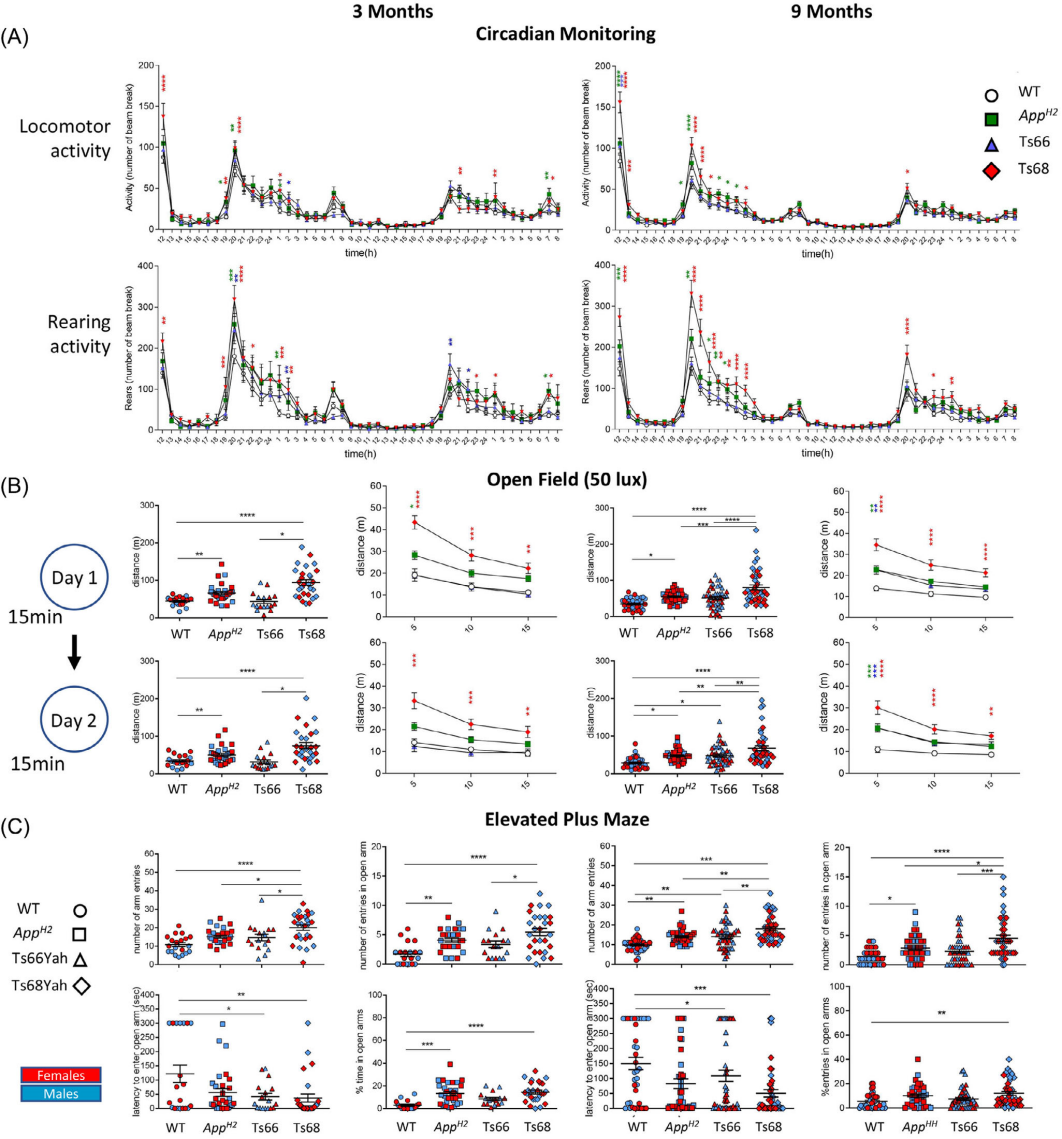
Behavioral characterization of 3‐month‐old and 9‐month‐old Ts68Yah DS model. (A) Increased locomotor activity and rears in Ts68Yah and *App^H2^* in CA test; (B) hyperactivity in Ts68Yah mice in OF; (C) higher exploration of open arm of EMP indicating increase open arm entries, reduced latency to enter the open arm and increased percent of time in the open arms in Ts68Yah mice; Both male (blue) and female (red) mice analysed. Statistics: for CA and OF two‐way rmANOVA, Tukey posthoc (*App^H2^* vs. WT *p < 0.05; **p < 0.001, ***p < 0.001, ****p < 0.0001; Ts66Yah vs. WT +*p* < 0.05 WT +*p* < 0.01+++*p* < 0.001; ++++*p* < 0.0001; Ts68Yah vs. WT # *p* < 0.05 ## *p* < 0.01; ### *p* < 0.001, ####*p* < 0.0001); for EPM two‐way ANOVA, Tukey post hoc (*p < 0.05; **p < 0.001, ***p < 0.001; ****p < 0.0001); one sample t‐test with theoretical mean value 0.50 for for NOR and 50 for Y‐maze (%SPA). Details of the mouse cohorts are provided in the Table S1. ANOVA, analysis of variance; CA, circadian activity; EPM, elevated plus maze; NOR, new object recognition; OF, open field; rmANOVA, repeated‐measures ANOVA.

A marked increase in locomotor and rearing activity was observed in Ts68Yah mice during the habituation phase and the dark cycle of the CA test (Figure 1A), consistent across both age groups. A similar, but less pronounced, increase in activity was observed in *App^H2^* mice. In the OF test (Figure 1B), at 3 months of age, Ts68Yah and *App^H2^* mice traveled significantly greater distances than WT and Ts66Yah mice on Day 1, with all genotypes showing reduced activity on Day 2. At 9 months of age, the pattern persisted, although Ts68Yah mice were no longer significantly more active than *App^H2^*. This hyperactivity at 3 months of age appeared to be driven by Aβ humanization, with an additional age‐related effect in Ts68Yah mice at 9 months of age.

In the EPM (Figure 1C), Ts68Yah and *App^H2^* mice showed increased arm entries at 3 months of age, including more frequent entries and longer durations in the open arms compared with WT controls, indicating reduced anxiety‐like behavior. Latency to enter the open arms was decreased in both Ts66Yah and Ts68Yah mice at 3 and 9 months of age. Hyperactivity persisted at 9 months of age, with Ts68Yah mice remaining the most active. All anxiety‐related metrics, open arm entries, latency, and percentage of time spent, were significantly elevated in Ts68Yah mice, suggesting a reduction in anxiety compared to other genotypes. These data indicate that humanization of Aβ causes a generalized elevation of activity and a decrease in anxiety in both euploid and DS models. In some contexts, these effects are magnified by the presence of the minichromosome.

**FIGURE 2:**
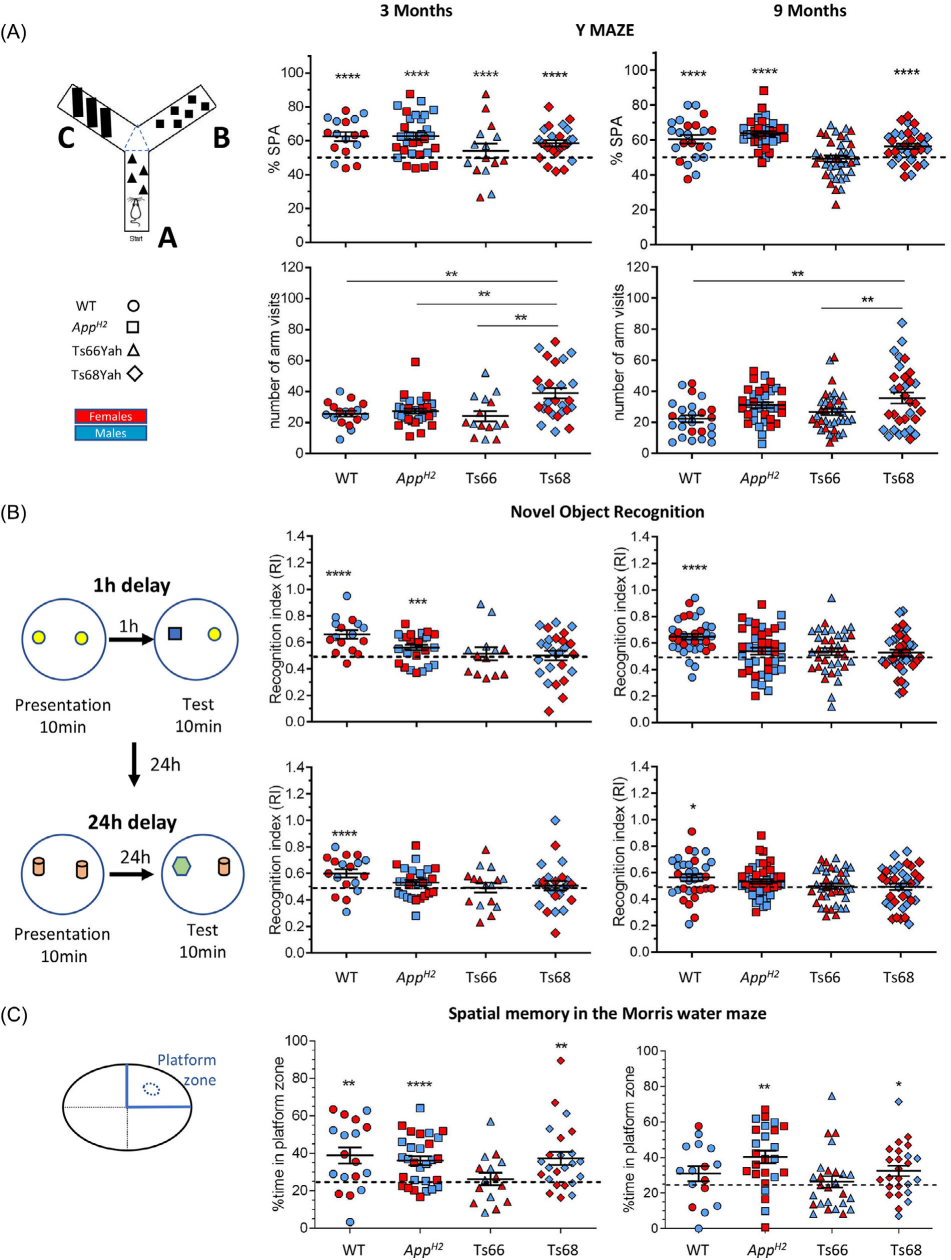
Cognitive profiling of 3‐month‐old and 9‐month‐old Ts68Yah DS model. (A) Ts68Yah mice show normal working memory in the Y‐maze test. (B) Impaired recognition memory in Ts68Yah and Ts66Yah mice in the NOR test with 1 and 24 h retention time. (C) Assessing spatial memories in the Ts68Yah mouse model. Both male (blue) and female (red) mice were analysed. Statistics: for Y‐maze (visits) 2‐way ANOVA, Tukey posthoc (*p < 0.05; **p < 0.001, ***p < 0.001; ****p < 0.0001); one‐sample t‐test with theoretical mean value 0.50 for NOR and 50 for Y‐maze (%SPA).Details of the mouse cohorts are provided in the Table S1. ANOVA, analysis of variance; DS, Down syndrome; NOR, new object recognition.

Working memory deficits in the Y‐maze were observed only in Ts66Yah mice at 9 months of age (Figure 2A), while Ts68Yah and *App^H2^* mice maintained normal spontaneous alternation. Ts68Yah mice exhibited increased arm visits at both ages, consistent with the hyperactivity observed in other tasks. This suggests that preserved alternation in Ts68Yah may result from increased activity rather than genuine rescue of minichromosome‐associated memory deficits.

We used the MWM to further test the effect of the minichromosome and Aβ humanization on learning and memory. During the learning phase, no differences in swimming speed were observed between genotypes (Figure S2). Consistent with our findings in the Y‐maze spontaneous alternation task, we also found that Ts66Yah mice were impaired in the probe trial of the MWM (24 h retention) at 3 months of age. In contrast, we observed no comparable probe trial deficits in Ts68Yah mice. Interestingly, the *App^H2^* mice performed better than the WT at 9 months of age.

To further investigate memory deficits, we studied short‐term (1 h retention) and long‐term (24 h retention) object recognition memory, using the NOR task. Both Ts66Yah and Ts68Yah were impaired in this task (Figure 2B) at 3 months and 9 months of age. In *App^H2^* mice, short‐term object memory was intact at 3 months but impaired at 9 months of age, suggesting that ageing may impact the effect of this genetic change. Overall, in some contexts, humanization of Aβ partially rescued spatial memory deficits associated with the Ts66Yah minichromosome.

To assess genotype and sex effects, a multivariate analysis was performed using 48 behavioral variables from both cohorts (Table S3), analyzed via the GDAPHEN pipeline.^51^ As shown in Figure S3, the four genotypes were separated along the first three dimensions, accounting for 33.2% of the total variance. Aβ humanization contributed significantly to the differences between genotypes, with a clear separation of *App^H2^* and Ts68Yah from the WT and Ts66Yah groups. Despite some overlap, *App^H2^* and Ts68Yah individuals were also dispersed by genotype. The ageing trajectory revealed that OF and anxiety‐related variables at 3 months were key contributors to the separation of genotypes in dimension 1. Overall, Ts68Yah mice exhibited more severe behavioral phenotypes compared to Ts66Yah, with increased activity across all tests, and early signs of reduced anxiety compared to the Ts66Yah DS model in which Aβ was not humanized.

### 3.3 Hippocampal intracellular accumulation of APP cleavage products and concurrent perturbation of endolysosomal pathways in the Ts68Yah mouse model of DSAD

**FIGURE 3:**
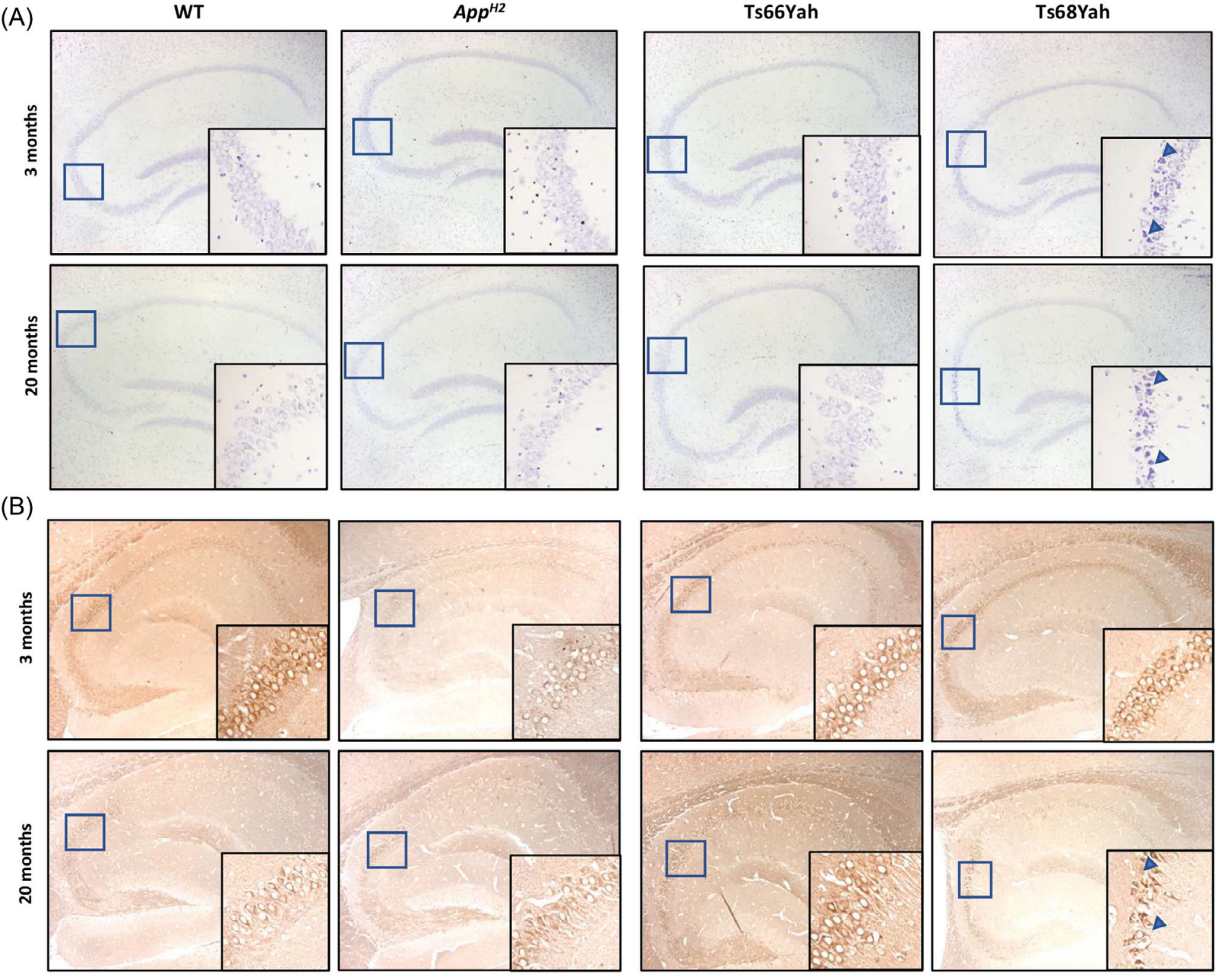
Morphological changes in the hippocampus of Ts68 mice at 3 and 20 months of age. Nissl staining on 5 µm paraffin‐embedded sagittal section at 3 and 20 months of age (A). Immunolocalization of APP with the 22C11 APP N‐ter antibody on a 5 µm paraffin‐embedded sagittal brain section (B). APP, amyloid precursor protein.

To understand the underlying cellular and molecular changes that contribute to behavioral alterations caused by Aβ humanization, we carried out a histological analysis of trisomic mice expressing a humanized form of Aβ, *App^H2^* (*n* = 3), Ts68Yah (*n* = 4), compared to the non‐humanized controls, WT (*n* = 3) and Ts66Yah (*n* = 3). We observed degenerating neurons in the pyramidal cell layer of the hippocampi of the Ts68Yah mouse at 3 months of age and later at 20 months of age by Nissl staining, that was not evident in other genotypes (Figure 3A). We found intracellular full‐length APP (22C11 N‐terminal) in the pyramidal cell layer of the hippocampus in all genotypes of mice, consistent with the known trafficking of the protein (Figure 3B). In addition, intracellular accumulation of cleaved APP (82E1, C‐terminal fragment‐β or Aβ) was observed in some pyramidal cells in the Ts68Yah hippocampus and cortex at 3 and 20 months of age (Figure S4). However, no extracellular Aβ deposits were detected.

**FIGURE 4:**
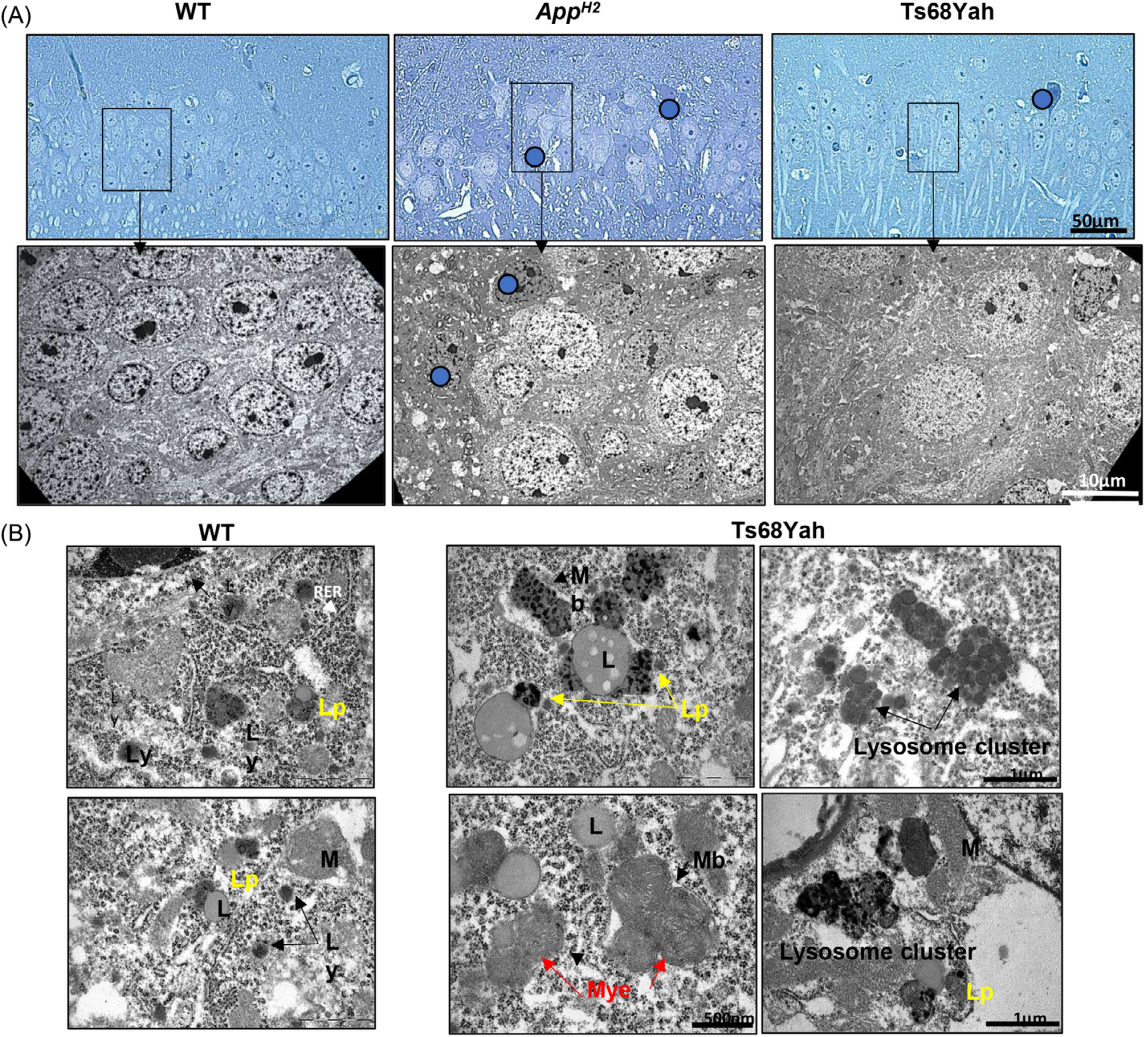
Ultrastructure of hippocampal cells in control and mutant mice at 9 months of age (A), with details of fine ultrastructure in the hippocampus of Ts68Yah mice (B). (A) Top: histological micrograph showing semi‐thin sections (2 µm) of pyramidal neurons in the hippocampus of 9‐month‐old male mice from WT, *App^H2^*, and Ts68Yah models. Note the presence of degenerating neurons (blue circle) in *App^H2^* mice and the severe decrease in neurons in Ts68Yah mice. Bottom: An electron micrograph of pyramidal neurons in the hippocampus showed the same phenotype. (B) High‐power view of lipofuscin granules (Lp. A, B, and D), myelinosomes (Mye. E), and lysosome cluster (C and F) from the same specimen as Figure 1. Note the characteristic granular electron‐dense contents and the lipid droplets (L) in lupofuscin. Note the myelinosomes found in the neurones of the hippocampus of Ts68Yah male mice, single‐membrane‐bound (Mb) myelinosomes containing multicentric myelin. Ly, lysosomes, M, mitochondria; RER, rough endoplasmic reticulum; WT, wild‐type.

To verify the changes in hippocampal neurons observed in the Ts68Yah (*n* = 4), we undertook transmission electron microscopy (TEM) on 9‐month‐old male hippocampus, compared to WT and *App^H2^* controls (*n* = 2). Consistent with our previous results, Ts68Yah mice showed a severe reduction in pyramidal neuronal density compared with WT animals (Figure 4A top panel), and degenerating cells were observed in both *App^H2^* and Ts68Yah samples. Ultrastructure of WT neurons was typical, with cytoplasm rich in mitochondria, lysosomes of different sizes, and lipofuscin granules (lipid‐associated lysosomes) (Figure 4B). In contrast, the Ts68Yah mice showed a very pronounced phenotype, with a reduced layer of pyramidal neurons compared to other *App^H2^* mice and WT, and significant cell death of neurons. In Ts68Yah samples, there was a substantial increase in lipofuscin granules and abnormal structures not observed in WT controls, including lysosome aggregates surrounded by a single membrane and myelinosomes, containing stacked, reticulated membranes (zebra‐like) (Figure 4B). This ultrastructure study pointed to an altered endolysosomal pathway in Ts68Yah hippocampal neurons.

### 3.4 Age‐dependent modification of APP processing and Aβ abundance in mouse models of DSAD

**FIGURE 5:**
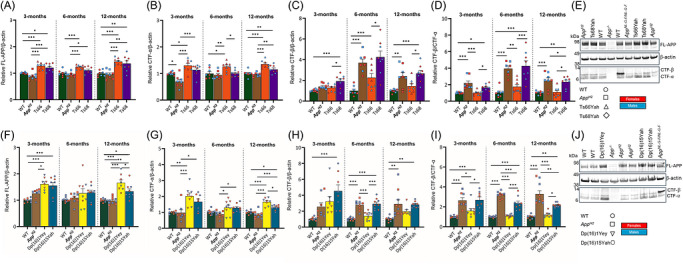
Quantification of cortical FL‐APP, APP CTF in the Ts68Yah mouse model. Abundance of FL‐APP (A, F), CTF‐α (B, G), CTF‐β (C, H), and the CTF‐β/CTF‐α ratio (D, I) were quantified in the cortex in WT (green), *App^H2^* (brown), Ts66Yah (orange), and Ts68Yah (purple) (A‐E) and WT (green), *App* ^H2^ (brown), Dp(16)1Yey (yellow), and Dp(16)15Yah (teal) (F‐I) mice at 3, 6, and 12 months of age by Western blot. Representative image of Western blot of 3 months of age in Ts68Yah and controls (E) and Dp(16)15Yah and controls (J). FL‐APP abundance (A) was affected by amyloid‐β humanization at 3 months (F(1,24) = 5.294, *p* = 0.03), 6 months (F(1,23) = 7.663, *p* = 0.011) and the presence of the min‐chromosome at 3 months (F(1,24) = 52.919, *p* < 0.001), 6 months (F(1,23) = 36.037, *p* < 0.001), and 12 months (F(1,24) = 41.095, *p* < 0.001) of age. CTF‐α abundance (B) was affected by amyloid‐β humanization at 3 months (F(1,24) = 10.563, *p* = 0.003), 6 months (F(1,23) = 7.947, *p* = 0.01), 12 months (F(1,24) = 7.474, *p* = 0.012) and by presence of the minichromosome at 3 months (F(1,24) = 26.877, *p* < 0.001), 6 months (F(1,23) = 8.544, *p* = 0.008) and 12 months (F(1,24) = 37.998, *p* < 0.001) of age. CTF‐β abundance (C) was affected by amyloid‐β humanization at 3 months (F(1,24) = 12.871, *p* = 0.001), 6 months (F(1,23) = 36.004, *p* < 0.001), 12 months (F(1,24) = 25.128, *p* < 0.001), and by presence of minichromosome at 3 months (F(1,24) = 7.695, *p* = 0.011), 6 months (F(1,23) = 6, *p* = 0.022) of age. The CTF‐β/CTF‐α ratio (D) was affected by amyloid‐β humanization at 3 months (F(1,24) = 28.443, *p* < 0.001), 6 months (F(1,23) = 46.512, *p* < 0.001) and 12 months F(1,24) = 37.744, *p* < 0.001) of age. FL‐APP abundance (F) was affected by the segmental duplication at 3 months (F(1,24) = 26.801, *p* < 0.001), 6 months (F(1,29) = 6.627, *p* = 0.015), 12 months F(1,27) = 44.607, *p* < 0.001) and amyloid‐β humanization at 12 months (F(1,27) = 4.361, *p* = 0.046) of age. CTF‐α abundance (G) was affected by segmental duplication at 3 months (F(1,24) = 25.87, *p* < 0.001), 6 months (F(1,29) = 17.663, *p* < 0.001), 12 months (F(1,27) = 66.812, *p* < 0.001), and amyloid‐β humanization at 12 months (F(1,27) = 14.007, *p* < 0.001) of age. CTF‐β abundance (H) was affected by amyloid‐β humanization at 3 months (F(1,24) = 5.813, *p* = 0.024), 6 months (F(1,29) = 82.629, *p* < 0.001), 12 months (F(1,27) = 15.845, *p* < 0.001); and the segmental duplication at 3 months (F(1,24) = 12.711, *p* = 0.002) of age. The CTF‐β/CTF‐α ratio (I) was affected by amyloid‐β humanization at 3 months (F(1,24) = 30.378, *p* < 0.001), 6 months (F(1,29) = 205.217, *p* < 0.001), 12 months (F(1,27) = 55.619, *p* < 0.001); and the segmental duplication at 6 months (F(1,29) = 9.943, *p* = 0.004) and 12 months (F(1,27) = 4.248, *p* = 0.049) of age, with a significant interaction between the effect of amyloid‐β humanization and the segmental duplication at 6 months (F(1,29) = 14.354, *p* < 0.001) and 12 months (F(1,27) = 8.899, *p* = 0.006) of age. ANOVA with factors of amyloid‐β humanization, presence of minichromosome or segmental duplication, and sex. Pairwise comparisons with Bonferroni correction for multiple comparisons, *p* < 0.05 *, *p* < 0.01 **, *p* < 0.001 ***. Error bars SEM. Data‐points are biological replicates, female (red), male (blue), sex was included as a variable in the ANOVA (reported in Table S4). *App* ^tm1Dbo/tm1Dbo^ (*App^−/−^*) samples were used as a negative control and *App* ^NL‐G‐F/NL‐G‐F^ samples as a positive control ^50^. ANOVA, analysis of variance; APP, amyloid precursor protein; CTF, C‐terminal fragment; FL, full length; WT, wild‐type.

To determine if changes to APP processing contributed to the perturbations in neuronal biology in the Ts68Yah model, we quantified the abundance of full‐length APP (FL‐APP) and its cleavage products APP‐C‐terminal fragment‐α (CTF‐α) and APP‐C‐terminal fragment‐β (CTF‐β) at 3, 6, and 12 months of age by Western blotting. The minichromosome increased FL‐APP and CTF‐α abundance in the cortex and hippocampus of Ts66Yah and Ts68Yah (Figure 5), consistent with the additional copy of *App* carried in these models (Figures 5A, B, S5A). Moreover, humanization of Aβ reduced CTF‐α abundance in the hippocampus at 3 months of age, in the cortex at all ages in *App^H2^* and Ts68Yah mice (Figures 5B and S5B), and increased CTF‐β abundance in Ts68Yah compared to Ts66Yah mice in the cortex at all ages, and in the hippocampus at 12 months of age (Figures 5C and S5C). Consistent with previous reports,^41^, ^42^, ^52^, ^53^ this led to an increase in CTF‐β/CTF‐α ratio in the brains of mice with humanized Aβ across the life span (Figures 5D and S5D). Furthermore, at 12 months of age, in both the hippocampus and cortex, the CTF‐β/CTF‐α ratio was reduced in Ts68Yah mice compared with *App^H2^* controls, indicating a modest age‐dependent effect of the minichromosome on APP processing. The sex of the mice did not affect the abundance of FL‐APP, CTF‐α or CTF‐β in the Ts68Yah study (Tables S4 and S5). To determine if humanization of the Aβ region resulted in detectable Aβ extracellular plaque deposition immunofluorescence for 82E1, a human Aβ N‐terminal specific antibody was used. At 12‐months of age, no significant formation of 82E1+ extracellular Aβ plaques was detected in either the hippocampus or cortex at 12‐months of age in either the *App^H2^* or Ts68Yah models (Figure S6).

To verify the effect of Hsa21‐orthologous additional gene content on APP processing observed in the Ts68Yah model, we undertook a similar study on the Dp(16)15Yah mouse model of DSAD (Figure 5F‐J). We found similar effects of *App* copy number and Aβ humanization on APP and cleavage product abundance in this alternative model. Moreover, as in the Ts68Yah model, the CTF‐β/CTF‐α ratio was reduced in Dp(16)15Yah mice compared with *App^H2^* controls in an age‐dependent manner (Figures 5I and S7). The sex of the mice did not affect the abundance of FL‐APP, CTF‐α, or CTF‐β in the Dp16(16)15Yah study (Tables S4 and S5).

**FIGURE 6:**
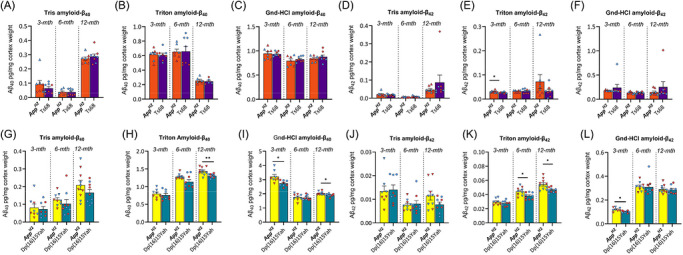
Quantification of cortical amyloid‐β_40_ and amyloid‐β_42_ in partially humanized mouse models. Abundance of (A‐C, G‐I) amyloid‐β_40_ and (D‐F, J‐L) amyloid‐β_42_ in the Tris soluble (A, D, G, J), 1% Triton soluble (B, E, H, K), and 5 M guanidine hydrochloride soluble (C, F, I, L) cortical tissue fractions WT (green), *App^H2^* (brown), Ts66Yah (orange), and Ts68Yah (purple) (A‐f) and WT (green), *App* ^H2^ (brown), Dp(16)1Yey (yellow), and Dp(16)15Yah (teal) (G‐L) mice at 3 months, 6 months, and 12 months of age were quantified by Meso Scale Discovery assay (6E10 amyloid‐β triplex). Ts66Yah, Dp(16)1Yey and WT controls were often below the limit of detection (Table S1) and thus excluded from analysis, such that Ts68Yah and Dp(16)2Yah were compared to *App^H2^* controls, using ANOVA with factors of genotype and sex. In the 1% Triton soluble fraction, Ts68Yah samples had reduced abundance of amyloid‐β_4_ (E) at 3 months of age (F(1,12) = 6.031, *p* = 0.03) compared to *App* ^H2^. In the 1% Triton soluble fraction, Dp(16)2Yah samples had reduced amyloid‐β_40_ at 6 months (F(1,14) = 7.014, *p* = 0.019) and 12 months (F(1,15) = 9.119, *p* = 0.009) of age (H), and amyloid‐β_42_ at 12 months of age (F(1,15) = 8.101, *p* = 0.012) (K) compared to *App^H2^*.In the 5 M guanidine hydrochloride insoluble fraction, the Dp(16)15Yah samples had reduced amyloid‐β_40_ at 3 months (F(1,12) = 7.563, *p* = 0.018) and 12 months (F(1,15) = 6.933, *p* = 0.019) of age (I); and amyloid‐β_42_ at 3 months of age (F(1,12) = 8.003, *p* = 0.015) (L) compared to *App^H/H^*. Pairwise comparisons with Bonferroni correction for multiple comparisons, *p* < 0.05 *, *p* < 0.01 **, *p* < 0.001 ***. Error bars SEM. Data‐points are biological replicates, female (red), male (blue), sex was included as a variable in the ANOVA (reported in Table S4). *App^tm1Dbo/tm1Dbo^* (*App^−/−^*) samples were used as a negative control and *App^NL‐G‐F/NL‐G‐F^* samples as a positive control (data not shown). ANOVA, analysis of variance; APP, amyloid precursor protein; WT, wild‐type.

We then determined whether the changes in APP processing affected the abundance or aggregation state of Aβ_40_ and Aβ_42_ in the brain by biochemical fractionation and quantification using the Mesoscale Discovery 6E10 assay. In *App^H2^* and Ts68Yah samples, humanization of Aβ resulted in detectable human Aβ in all fractions at all ages (Figure 6). In contrast, human Aβ was not detectable in other genotypes (WT and Ts66Yah) that only produce mouse Aβ. In the Ts68Yah cohort at 3 and 6 months of age, sex affected the abundance of Tris‐soluble Aβ_40_ and Aβ_42,_ and at 6 months of age, sex also effected the abundance of membrane‐associated Aβ_40_ and Aβ_42_ (Table S6). At 6 months of age in the membrane‐associated fraction, an interaction of sex and the minichromosome was found to affect Aβ_42_ abundance such that a difference in abundance was detected between samples from *App^H2^* and Ts68Yah females but not males, no other interactions of sex and genotype were observed (Table S4). In the Ts68Yah model, a decrease in membrane‐associated Aβ_42_ abundance (Figure 6E) compared to *App^H2^* controls occurred at 3 months of age. Consistent with this, in the Dp(16)15Yah model compared to *App^H2^* controls, a decrease in aggregated Aβ_40_ and Aβ_42_ at 3 months of age, membrane‐associated Aβ_42_ at 6 and 12 months of age, and membrane‐associated and aggregated Aβ_40_ at 12 months of age occurred (Figure 6I, K, L). No effect of sex on Aβ abundance was found in this study (Supplementary table 4). In summary, the additional gene content carried in the DSAD models results in a small reduction in Aβ abundance consistent with the age‐dependent decrease in CTF‐β/CTF‐α ratios.

### 3.5 Tau biology and autophagy markers alterations in the Ts68Yah mouse model of DSAD

**FIGURE 7:**
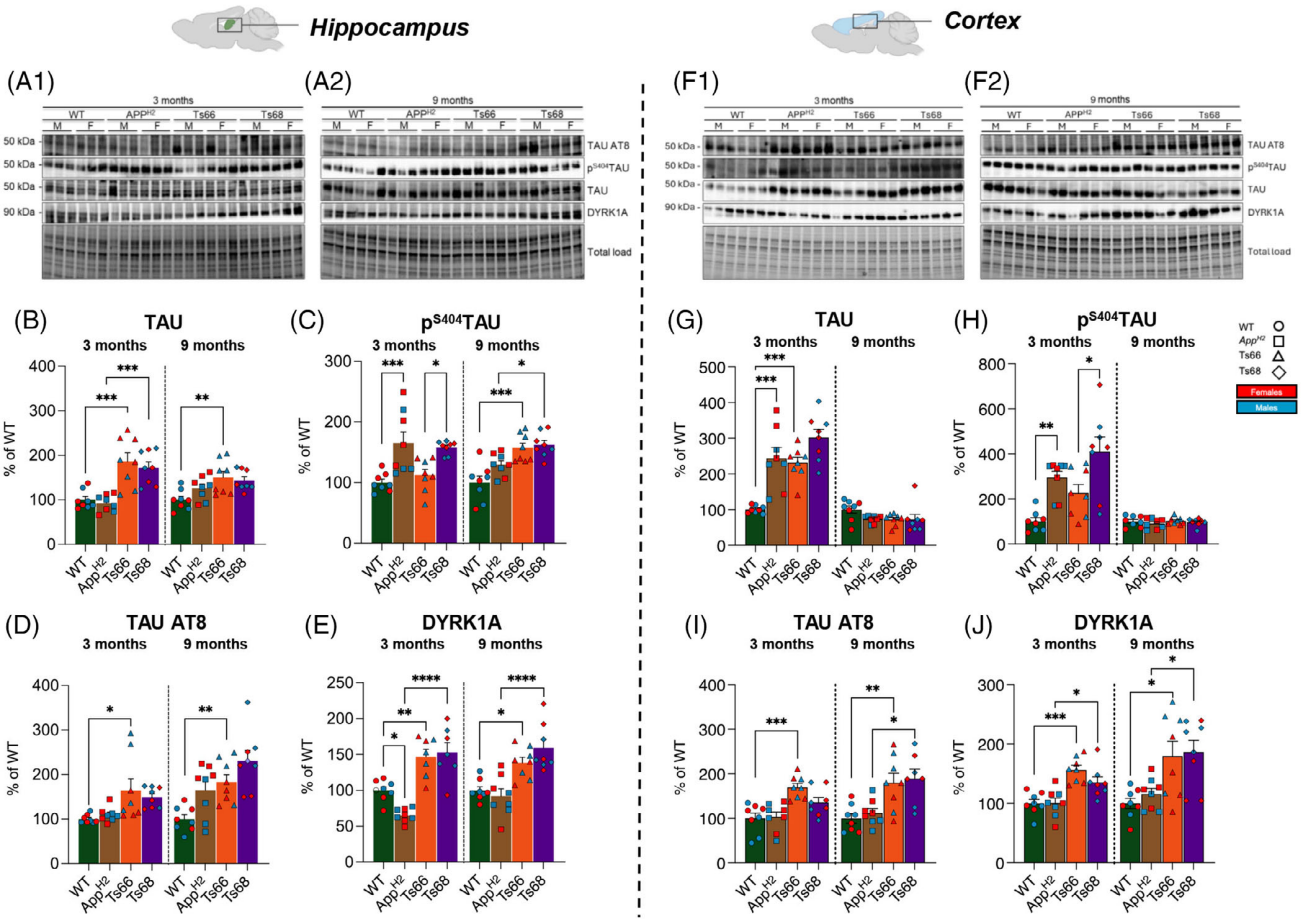
Impact of neurodegenerative and antioxidant molecular signatures in Ts66 and Ts68 hippocampus and cortex. (A1) and (A2) Representative Western blots and densitometric evaluation of total Tau (B, H), phosphorylation of Tau on respectively Ser404 residue (C, I) and Ser202/Thr205 residues (AT8) (D, J) and total DYRK1A (E, K) in the hippocampus and cortex of mice at different ages 3 months (WT *n* = 8, *App^H2^ n* = 8, Ts66Yah *n* = 8, Ts68Yah *n* = 8) and 9 months (WT *n* = 8, *App^H2^* n = 8, Ts66Yah *n* = 8, Ts68Yah *n* = 8). (B) Hippocampus levels of Tau: Ts66Yah mice vs. WT at 3 months of age (+86%, *p* = 0.0002; F(1,28) = 4.77) and 9 months of age (+50%, *p* = 0.006; F(1,28) = 3.52), Ts68Yah mice vs. *App^H2^* at 3 months of age (+79%, *p* = 0.0006; F(1,28) = 4.36). (C) Tau^S404^ phosphorylation: *App^H2^* vs. WT 3 months (+65%, *p* = 0.0009; F(1,28) = 4.24), Ts68Yah vs. Ts66Yah 3 months (+45%, *p* = 0.02; F(1,28) = 2.95), in 9 months animal Ts66Yah vs, WT (+57%, *p* = 0.0002; F(1,28) = 4.81) and in Ts68 vs. *App^H2^* (+33%, *p* = 0.04; F(1,28) = 2.76). (D) Tau AT8 phosphorylation: Ts66Yah vs. WT at 3 months of age (+56%, *p* = 0.047; F(1,28) = 2.69) and at 9 months of age(+83%, *p* = 0.02; F(1,28) = 3.49). (E) DYRK1A protein levels: at 3 months of age, *App^H2^* vs. WT (+34%, *p* = 0.02; F(1,28) = 3.08), Ts66 vs. WT (+47%, *p* = 0.0001; F(1,28) = 4.15) and Ts68 vs. *App^H2^* (+88%, *p* = 0.0001; F(1,28) = 7.76). (G) Tau expression in cortex: Ts66Yah vs. WT 3 months (+130%, *p* = 0.0005; F(1,28) = 4.46); *App^H2^* vs. WT 3 months (+143%, *p* = 0.0001; F(1,28) = 4.90). (H) Tau^S404^ phosphorylation: *App^H2^* vs. WT 3 months (+195% *p* = 0.009; F(1,28) = 3.35) and Ts68Yah vs. Ts66Yah 3 months (+182%, *p* = 0.01; F(1,28) = 3.13). (I) Tau^S202/T205^ AT8 phosphorylation: Ts66Yah vs. WT at 3 months (+69%, *p* = 0.0004; F(1,28) = 4.53) and at 9 months (+80%, *p* = 0.007; F(1,28) = 3.46), and Ts68Yah vs. *App^H2^* 9 months (+77%, *p* = 0.01; F(1,28) = 3.23). (J) DYRK1A protein levels: Ts66Yah vs. WT mice 3 months (+56%, *p* = 0.0001; F(1,28) = 5.01), Ts68Yah vs. *App^H2^* 3 months (+35%, *p* = 0.01; F(1,28) = 3.08); At 9 months Ts66Yah vs. WT mice (+80%, *p* = 0.01; F(1,28) = 3.28) and Ts68Yah vs. *App^H2^* (+71%, *p* = 0.02; F(1,28) = 2.91). Expression and phosphorylation levels were normalized to the total protein load. All densitometric values are given as a percentage of WT at respectively 3 months and 9 months set as 100%. Data are presented as means ± SEM. Statistical significance was determined using ANOVA with Bonferroni's multiple comparisons test (*p < 0.05, **p < 0.01, ***p < 0.001, ****p < 0.0001). Columns were used to show differences among the groups (WT vs. Ts66Yah, *App^H2^* vs. Ts68Yah, WT vs. *App^H2^*, and Ts66Yah vs. Ts68Yah). Dots were used for WT, squares for *App^H2^* triangles for Ts66Yah, and rhombuses for Ts68Yah. Blue color indicates male mice (n = 4) and red color indicates female mice (n = 4). ANOVA, analysis of variance; APP, amyloid precursor protein; SEM, standard error of the mean; WT, wild‐type.

To further evaluate the mechanism underlying changes to neuronal biology in the Ts68Yah model, we analyzed Tau protein abundance and phosphorylation at S404 and Ser202/Thr205 (AT8) residues (Figure 7). We found that the minichromosome elevated total Tau protein at 3 months of age, and phospho‐Tau^S202/205^ at 3 and 9 months of age, in both the hippocampus and cortex. Whereas humanization of Aβ was associated with an elevation of phospho‐Tau^S404^ in the cortex at 3 months, at 9 months of age this was exacerbated by the minichromosome, such that the abundance of phospho‐Tau^S202/205^ was increased in the cortex and phospho‐Tau^S404^ in the hippocampus in Ts68Yah compared with *App^H2^* controls (Figure 7B‐C, G‐I). The Hsa21‐encoded kinase DYRK1A is implicated in changes to Tau phosphorylation in DSAD, and *Dyrk1A* is found in three copies in the Ts66Yah and Ts68Yah models, resulting in elevated DYRK1A protein in both hippocampus and cortex at 3 and 9 months of age, which may contribute to the observed differences in phospho‐Tau observed in Ts66Yah and Ts68Yah models. (Figure 7E, J). The analysis of the Hsa21‐encoded protein SOD1 in the hippocampus and cortex of Ts66Yah and Ts68Yah confirms the influence of the minichromosome on protein profile changes (Figure S8)

**FIGURE 8:**
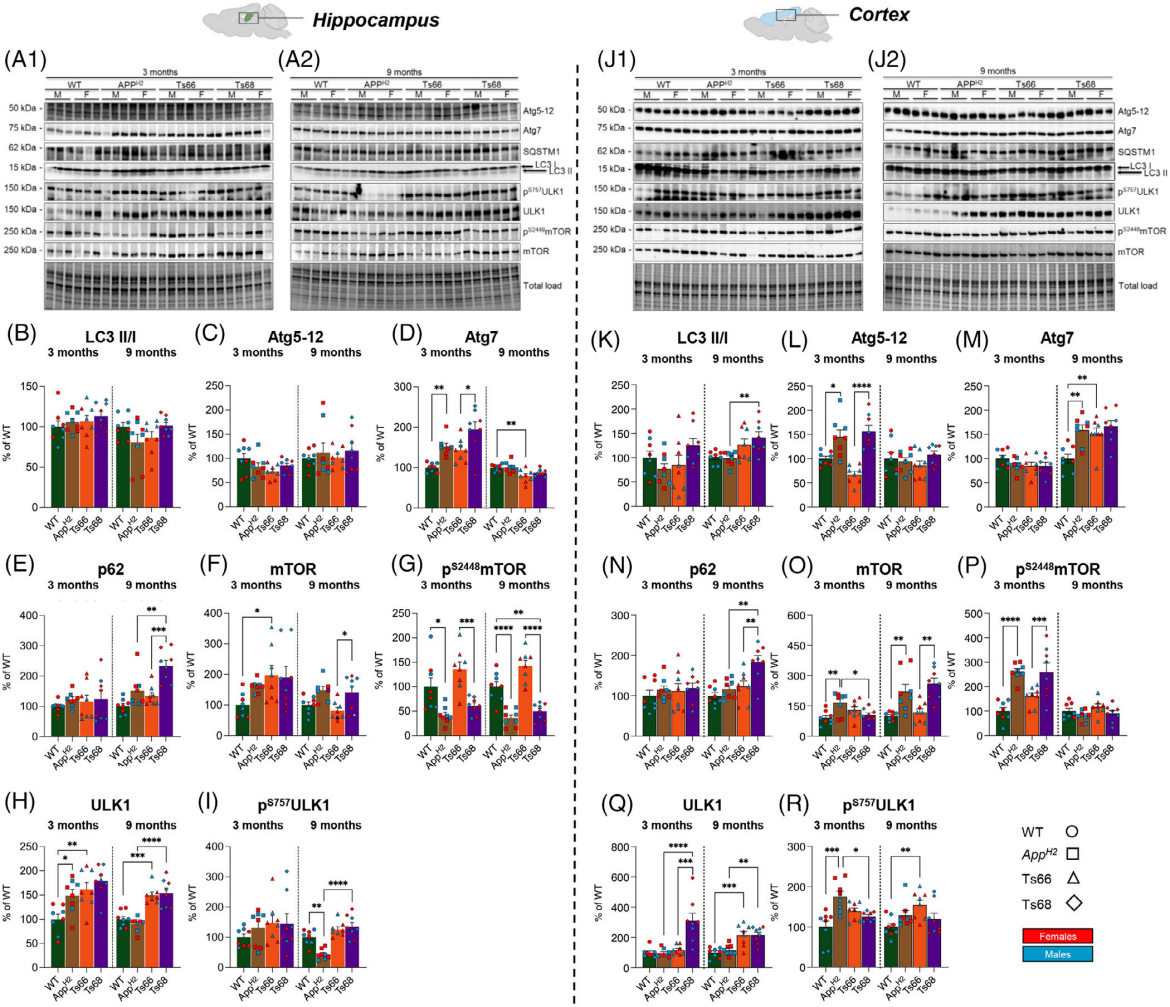
Tissue‐dependent analysis of key autophagy regulators in the hippocampus and cortex of Ts66Yah and Ts68Yah mouse models. (A1) and (A2) Representative Western blots and densitometric evaluation of LC3 II/I ratio (B, K), Atg5‐12 (C, L), Atg7 (D, M), p62 (or SQSTM1) (E, N), total level of mTOR (F, O), phosphorylation of mTOR on Ser2448 residue (G, P), total levels of ULK1 (H, Q), and phosphorylation of ULK1 on Ser757 residue (I, R) in the hippocampus and cortex of mice at different ages 3 months (WT n = 8, *App^H2^* n = 8, Ts66Yah n = 8, Ts68Yah n = 8) and 9 months (WT n = 8, *App^H2^* n = 8, Ts66Yah n = 8, Ts68Yah n = 8). (B) LC3 II/I ratio in the hippocampus: no significant alteration observed. (C) Atg5‐12 protein levels: no significant alteration observed. (D) Atg7 levels: at 3 months of age *App^H2^* vs. WT (+52%, *p* = 0.01; F(1,28) = 3.19) and Ts68Yah vs. Ts66Yah (+51%, *p* = 0.02; F(1,28) = 3.10); Ts66Yah vs. WT at 9 months (‐21%, *p* = 0.007; F(1,28) = 3.45). (E) p62 levels: Ts68Yah vs. *App^H2^* at 9 months (+82%, *p* = 0.003; F(1,28) = 3.78) and Ts68Yah vs. Ts66Yah at 9 months (+98%, *p* = 0.0004; F(1,28) = 4.56). (F) mTOR expression: Ts66Yah vs. WT at 3 months of age (+98%, *p* = 0.04; F(1,28) = 2.70) and Ts68Yah vs.Ts66Yah at 9 months (+60%, *p* = 0.02; F(1,28) = 3.00). (G) mTOR^S2428^ phosphorylation: at 3 months of age *App^H2^* vs. WT (‐58%, *p* = 0.01; F(1,28) = 3.28) and Ts68Yah vs. Ts66Yah (‐75%, *p* = 0.001; F(1,28) = 4.19); 9 months of age *App^H2^* vs. WT (‐63%, *p* = 0.0001; F(1,28) = 5.12) and Ts68Yah vs. Ts66Yah (‐92%, *p* = 0.0001; F(1,28) = 7.41). (H) ULK1 expression levels: 3‐month‐old animals *App^H2^* vs. WT (+49%, *p* = 0.03; F(1,28) = 3.56) and Ts66Yah vs. WT (+61%, *p* = 0.005; F(1,28) = 3.56); at 9‐month‐old animals Ts66Yah vs. WT (+50%, *p* = 0.001; F(1,28) = 5.01) and Ts68Yah vs. *App^H2^* (+64%, *p* = 0.0001; F(1,28) = 6.42). (I) ULK1^S757^ phosphorylation: 9 months of age *App^H2^* vs. WT (‐56%, *p* = 0.001; F(1,28) = 1.92) and Ts68Yah vs. *App^H2^* (+92%, *p* = 0.0001; F(1,28) = 6.67). (K) LC3 II/I ratio in the cortex: in 3‐month‐old Ts68Yah vs. *App^H2^* [+89%, *p* = 0.0001; F(1,28) = 6.10]. (L) Atg5‐12 levels: at 3 months of age *APP^H2^* vs. WT (+45%, *p* = 0.01; F(1,28) = 3.09) and Ts68Yah vs. Ts66Yah (+89%, *p* = 0.0001; F(1,28) = 6.10). (M) Atg7 protein levels: 9‐month‐old animals *App^H2^* vs. WT (+59%, *p* = 0.002; F(1,28) = 3.83) and Ts66Yah vs. WT (+52%, *p* = 0.009; F(1,28) = 3.35). (N) p62 protein levels: 9‐month‐old animals Ts68Yah vs. Ts66Yah (+61%, *p* = 0.004; F(1,28) = 3.6) and Ts68Yah vs. *App^H2^* (+67%, *p* = 0.001; F(1,28) = 4.03). (O) mTOR expression levels: 3 months of age Ts66Yah vs. WT (+60%, *p* = 0.03; F(1,28) = 2.8); 9‐month‐old mice *APP^H2^* vs. WT (+121%, *p* = 0.005; F(1,28) = 3.54) and Ts68Yah vs. Ts66Yah (+142%, *p* = 0.0012; F(1,28) = 4.14). (P) mTOR^S2448^ phosphorylation: 3‐month‐old animals *App^H2^* vs. WT (+162%, *p* = 0.0001; F(1,28) = 5.9) and Ts68Yah vs. Ts66Yah (+122%, *p* = 0.0007; F(1,28) = 4.35). (Q) ULK1 protein levels: 3‐month‐old animals Ts68Yah vs. *App^H2^* (+213%, *p* < 0.0001; F(1,28) = 5.5) and Ts68Yah vs.Ts66Yah (+193%, *p* = 0.0001 F(1,28) = 4.98); at 9 months Ts66Yah vs. WT (+115%, *p* = 0.0004; F(1,28) = 4.58) and Ts68Yah vs. *App^H2^* (+99%, *p* = 0.002; F(1,28) = 3.84). (R) ULK1^S757^ phosphorylation: 3‐month‐old animals *App^H2^* vs. WT (+75%, *p* = 0.001; F(1,28) = 4.96) and Ts68Yah vs. *App^H2^* (‐49%, *p* = 0.01; F(1,28) = 3.23); 9 months of age Ts66Yah vs. WT (+55%, *p* = 0.002; F(1,28) = 3.08). Protein levels were normalized to total protein load. All densitometric values are given as a percentage of WT at, respectively, 3 months and 9 months set as 100%. Data are presented as means ± SEM. Statistical significance was determined using one‐way ANOVA with Bonferroni's multiple comparisons test (*p < 0.05, **p < 0.01, ***p < 0.001, ****p < 0.0001). Columns were used to show differences among the groups (WT vs. Ts66Yah, *App^H2^* vs. Ts68Yah, WT vs. *App^H2^*, and Ts66Yah vs. Ts68Yah). Dots were used for WT, squares for *App^H2^*, triangles for Ts66Yah, and rhombuses for Ts68Yah. Blue color indicates male mice (n = 4) and red color indicates female mice (n = 4). ANOVA, analysis of variance; APP, amyloid precursor protein; WT, wild‐type

To further understand the disruption of endolysosomal pathways in the Ts68Yah model, as reported by TEM (Figure 4), we analyzed the abundance of key autophagy markers in the cortex and hippocampus by Western blot (Figure 8). With three‐way ANOVA analysis (Figure S9) for genotype, sex and age, we found an ageing‐dependent increase in LC3 II/I ratio in Ts68Yah cortex compared to *App^H2^* (Figure 8B), and raised p62 (SQSTM), a marker of faulty cargo degradation, in both hippocampus and cortex (Figure 8E, K and N); consistent with changes in neuronal intracellular structure in this model. Humanization of Aβ resulted in an increase in ATG5‐12 in the cortex at 3 months of age (Figure 8L) and ATG7 at 9 months of age (Figure 8M). In the hippocampus, humanization of Aβ was associated with raised ATG7 at 3 months of age (Figure 8D), but no differences in the abundance of these proteins between Ts68Yah and *App^H2^* controls were observed.

To further determine if changes to the regulation of the autophagy‐lysosomal degradation pathway contributed to the alterations in the Ts68Yah model, we analyzed the activation of one of the main autophagy repressors, mTOR, which is altered in DS and AD humans and mice ^34^, ^35^. Total mTOR abundance was raised by the humanization of Aβ in the cortex at 3 and 9 months of age (Figure 8O). Moreover, at 3 months of age, mTOR abundance was reduced in Ts68Yah compared with *App^H2^* controls in the cortex (Figure 8F). However, similar changes were not observed in the hippocampus in these genotypes (Figure 8F and G). Humanization of Aβ resulted in increased phospho‐mTOR^S2448^ in the cortex at 3 months of age, but not at 9 months, while a decrease in hippocampus at both 3 and 9 months of age (Figure 8G and P) was observed.

To further evaluate the effect of Aβ humanization on the autophagy‐regulatory role of mTOR, we analyzed ULK1 abundance and inhibitory phosphorylation (S757), a known target site of mTOR. Elevated abundance of total ULK1 and phospho‐ULK1^S757^, consistent with observed changes in mTOR, occurred in the cortex at 3 and 9 months of age. In the cortex, ULK1 was elevated in Ts68Yah compared to the *App^H2^* and Ts66Yah controls at 3 and 9 months of age, and a similar increase was observed in the hippocampus at 9 months of age (Figure 8H). Hippocampal phospho‐ULK1^S757^ was reduced in *App^H2^* animals at 9 months of age compared to WT, but levels were elevated in Ts68Yah, in line with the reduction of mTOR phosphorylation. Although increased mTOR S2448 phosphorylation characterizes ageing in DSAD, our models show region‐ and age‐dependent differences. Aβ humanization transiently elevates cortical p‐mTOR(S2448), but reduces hippocampal levels of this analyte. Autophagy impairment, as indicated by p62 accumulation and endolysosomal abnormalities, suggests defective cargo degradation rather than sustained mTOR‐driven repression, which diverges from late‐stage human DSAD mechanisms.

## 4 DISCUSSION

We report the first DSAD knock‐in Aβ‐humanized mouse models, which reproduce several early features of AD. Despite considerable effort, we were unable to establish lines carrying three partially humanized App copies together with additional Hsa21‐orthologous genes. Our findings indicate that this genotype is incompatible with long‐term survival on either the sighted B6C3B or C57BL/6J backgrounds.

The new DSAD model (Ts68Yah) displays cognitive impairment, increased activity, and risk‐taking behavior (Table S7), together with intracellular Aβ accumulation, tau phosphorylation, and disruptions to endolysosomal and autophagic pathways. DS models typically show learning and memory deficits, and here the humanized Aβ peptide altered responses in several tests, especially amplifying hyperactivity and anxiety‐related behaviors. Unexpectedly, Aβ humanization improved Y‐maze and MWM performance in Ts68Yah mice compared with Ts66Yah ^29^, ^30^. Similar effects have been reported in other Aβ humanized models,^41^, ^42^, ^52^, ^53^ suggesting that the three amino‐acid differences between human and mouse Aβ influence normal brain function.

Ts68Yah mice also made more visits and spent more time in the open arms of the EPM, consistent with increased risk‐taking, a behavior observed in some individuals with AD or mild cognitive impairment.^54^, ^55^, ^56^, ^57^ Whereas typical ageing tends to shift behavior toward caution, AD is associated with impaired judgement and altered risk assessment. Thus, our DSAD model offers a valuable tool for investigating and developing interventions targeting decision‐making and risk‐assessment changes associated with AD‐related cognitive impairment.

The intraneural accumulation of APP and endolysosomal dysfunction observed in Ts68Yah mice are key early mechanisms of DSAD that may accelerate disease progression.^58^, ^59^, ^60^ Endo‐lysosomal abnormalities are a hallmark of many neurodegenerative disorders and appear early in AD.^61^, ^62^ In DS models, these alterations arise from the combined effects of *App* triplication and other Hsa21 genes, including *Dyrk1a* and *Synj1*, which regulate vesicular trafficking, endocytosis, and endosomal dynamics.^63^ In this model, endosomal defects are accompanied by changes in autophagic markers, indicating disruption of degradative pathways, consistent with findings in human DS and AD brains.^17^, ^18^, ^19^, ^64^ However, in Ts68Yah mice, mTOR activation appears to contribute minimally to autophagy repression. Instead, autophagic impairment primarily affects phagosome maturation and p62‐dependent cargo degradation, promoting the accumulation of protein aggregates.

Protein trafficking therefore represents a critical, potentially modifiable pathway in AD and related disorders. In our study, Ts68Yah hippocampal neurons showed an enrichment of myelinosomes, vesicular structures typically forming under cellular stress when oligodendrocytes are damaged or when axons undergo degeneration and myelin breakdown. Beyond marking neurodegeneration, myelinosomes may contribute to proteostasis by exporting aberrant proteins rather than degrading them intracellularly. Altered myelination and increased vulnerability of the myelin–axon interface have been reported in AD,^65^, ^66^, ^67^ while delayed or reduced myelination is well documented in trisomy 21 and linked to intrinsic oligodendrocyte differences.^68^ Thus, myelinosome formation may directly reflect the degeneration of the subnormally myelinated neurons characteristic of DS. Overall, these findings suggest that myelinosomes represent a non‐degradative proteostasis route enabling neurons to expel misfolded or aggregated proteins under conditions of endolysosomal dysfunction ^69^. The Ts68Yah mouse therefore provides a valuable model for investigating these mechanisms and for testing interventions targeting endolysosomal defects, proteostasis, and myelination disturbances in DSAD biology.

Our data show that an additional copy of *App* in the Ts66Yah and Dp(16)1Yey mouse models leads to elevated FL‐APP and CTF‐α abundance in the cortex across the life span, consistent with reports of raised FL‐APP and CTF‐α in Ts65Dn and Dp(16)1Yey models^38^, ^70^, ^71^. In the hippocampus, this upregulation is less robust, suggesting region‐specific differences in the impact of *App* dosage. Recent label‐free proteomics of the Ts66Yah frontal cortex revealed no significant changes in the abundance of key APP‐processing enzymes, ADAM10, BACE1, or PSEN1, indicating that increased *App* gene dosage, rather than altered enzyme levels, primarily drives these changes ^72^.

Humanization of Aβ in the DS mouse models described here reduces FL‐APP and CTF‐α levels at specific time points. Both Ts68Yah and Dp(16)15Yah mice show lower fragment abundance than their respective controls (Ts66Yah and Dp(16)1Yey). In contrast, partial humanization of *App* increases CTF‐β levels across the life span in the cortex, and in the hippocampus at 6 and 12 months, consistent with euploid rodent studies ^43^. As previously reported, humanized *App* shifts the CTF‐β/CTF‐α ratio toward the β‐secretase pathway, favoring Aβ generation.

However, these new models also reveal a consistent age‐dependent reduction in the CTF‐β/CTF‐α ratio driven by the additional Hsa21‐orthologous gene content. This indicates that at least one triplicated Hsa21 gene orthologue partially suppresses Aβ production. Supporting this, we observe modest reductions in Aβ levels in Dp(16)15Yah mice and some evidence of reduced levels in Ts68Yah animals. These findings align with next‐generation DSAD mouse models in which triplication of the Dp3Tyb region or the presence of a human transchromosome reduces Aβ deposition.^24^

BACE2, a β‐secretase encoded on Hsa21, is a strong candidate for mediating this effect. The mouse orthologue *Bace2* is present in three copies in Ts68Yah, Dp(16)15Yah, and Dp3Tyb models, and BACE2 has been implicated in modifying Aβ pathology in trisomy 21 induced pluripotent stem cells (iPSC) ‐derived organoids.^73^ Nonetheless, some studies in adult mouse models and human post‐mortem DS brains have found no change in BACE2 abundance or activity, whereas increased BACE1 levels have been reported in females with DSAD and in female Dp(16)1Yey mice, suggesting possible sex‐specific interactions.^38^, ^46^, ^74^, ^75^ An alternative explanation is that residual mouse Aβ, produced from the non‐humanized *App* alleles in Ts68Yah and Dp(16)15Yah, interferes with membrane binding or aggregation of human Aβ. This mixed‐species environment could influence Aβ dynamics and contribute to the observed reductions. Overall, these data highlight how *App* humanization interacts with Hsa21 gene content to shape APP processing and Aβ biology in DSAD, underscoring the value of these new models for mechanistic and therapeutic studies.

The overexpression and hyperphosphorylation of Tau, particularly at serine and threonine residues, promote its self‐assembly into toxic neurofibrillary tangles (NFTs), a hallmark of AD‐like pathology.^9^ Our findings show that both trisomy and partial APP humanization contribute to increased tau phosphorylation in the frontal cortex and hippocampus of Ts68Yah mice. Data from these brain regions indicate a strong influence of genotype (trisomy and *App^H2^*), potentially interacting in both young and aged animals, whereas sex‐related effects appear minimal. As humanization of the Aβ sequence also influences Tau phosphorylation, this effect should be carefully considered when designing therapeutic strategies. In particular, reducing expression of the Aβ sequence may help mitigate downstream Tau‐related pathology. Consistent with previous observations in Ts66Yah mice, here we find elevated Tau levels in both trisomic and APP‐humanized mice at early stages. In Ts68Yah mice, Tau phosphorylation at the S404 residue increases early but does not persist into old age, when phosphorylation instead shifts to S202 and T205 sites (recognized by AT8 antibody). In DS murine models, phosphorylation at S404 and AT8 sites correlates with elevated DYRK1A levels.^76^ Our analysis confirms trisomy‐driven upregulation of DYRK1A in the frontal cortex and hippocampus. Here we find that DYRK1A overexpression appears to have age‐dependent effects on Tau phosphorylation, consistent with a previous report.^30^, ^77^ These observations suggest that late‐stage intervention using DYRK1A kinase inhibitors could offer a promising strategy to counteract Tau hyperphosphorylation in persons who have DS as AD progresses in these individuals.^78^

The knock‐in partially humanized *App* DSAD models presented here provide several advantages over previous DSAD mouse models. Classical DS mouse models, such as the Ts66Yah and Dp16(1)Yey, only generate mouse Aβ, and do not accumulate pathogenic aggregates of the peptide in the brain.^38^, ^39^ Similarly, the Tc(HSA21, CAG‐EGFP)1Yakaz mouse model with an additional copy of Hsa21, has also been reported not to accumulate pathogenic Aβ in the brain.^40^ Alternative approaches have crossed DS mouse models with transgenic or gene‐targeted mouse models of autosomal dominant AD (ADAD),^24^, ^40^, ^46^, ^79^, ^80^ to study DSAD mechanisms. However, this experimental approach is confounded by the use of causal mutations that cause the early development of AD rather than just increasing the *App* dosage as occurs in persons with DSAD.

The Ts68Yah and Dp(16)15Yah mouse models represent a major step forward in modeling DS‐associated early AD, as they recapitulate key pathological and behavioral alterations. Both models show increased activity, risk‐taking behavior, tau hyperphosphorylation, endolysosomal defects, and APP processing shifts toward the β‐secretase pathway. Humanization of Aβ further modulates behavior, improving specific cognitive performances while amplifying anxiety‐related traits. The presence of myelinosomes and impaired autophagic flux reinforces the relevance of Ts68Yah for studying early AD mechanisms. These results highlight how trisomy, APP dosage, and Aβ sequence interact to shape disease trajectories and position these models as valuable tools for mechanistic studies and therapeutic evaluation in DSAD. Nonetheless, limitations remain: the behavioral improvements after Aβ humanization may not fully mirror DSAD; mTOR signaling does not reproduce the persistent S2448 hyperactivation seen in ageing DSAD; myelinosomes have not been observed in human DS tissue; finally, the analysis of synaptic dysregulation, particularly age‐dependent alterations in dendritic spine morphology, a hallmark of DS neuropathology, remains an important avenue for future investigation. Integration with humanized genes and iPSC‐derived microglia will help address species‐specific differences.

## CONFLICT OF INTEREST STATEMENT

The authors declare that they have no competing interests. F.K.W. has undertaken for fee consultancy for Alnylam Pharmaceuticals and TRIMTECH Pharmaceuticals unrelated to the work in this report. Author disclosures are available in the supporting information.

## CONSENT STATEMENT

All human subjects provided informed consent.

## Supporting information




**Supporting Information**: alz71498‐sup‐0001‐ICMJE.pdf

**Supporting Information**: alz71498‐sup‐0002‐FiguresS1‐S9.pdf

**Supporting Information**: alz71498‐sup‐0003‐TableS1.docx

**Supporting Information**: alz71498‐sup‐0004‐TableS2.docx

**Supporting Information**: alz71498‐sup‐0005‐TableS3.docx

**Supporting Information**: alz71498‐sup‐0006‐TableS4.docx

**Supporting Information**: alz71498‐sup‐0007‐TableS5.docx

**Supporting Information**: alz71498‐sup‐0008‐TableS6.docx

**Supporting Information**: alz71498‐sup‐0009‐TableS7.docx
