# Supplementary material for: Age‐related behavioral and molecular landmarks in new mouse models for studying Alzheimer's disease in Down syndrome
Source: Alzheimers Dement. 2026 May 21;22(5):e71498. doi: 10.1002/alz.71498 (PMC13240120; doi:10.1002/alz.71498)

Age-related Behavioural and Molecular Landmarks in New Mouse Models for Studying Alzheimer's Disease in Down Syndrome

Authors:
Monika Rataj Baniowska[1,#], Paige Mumford[2,#], Francesca Prestia[3], Pauline Stephan[1], Millie Beament[2], Marie-Christine Birling[4], Chiara Lanzillotta[3], Letizia Ciafardini[3], Eugenio Barone[3], Gloria Lau[2], Claire Chevalier[1], Chadia Nahy[1], Nadia Messaddeq[1], Thais Lestra[2], Yixing Wu[2], Valérie Nalesso[1], Fabio Di Domenico[3,*], Frances Wiseman[2,*] and Yann Herault[1,4,5,*]
#equal first authors

Supplementary Figures

Figure S1: The humanised Aβ allele.
A comparison of the amino-acid sequence of the amyloid β peptide with the position of cleavage. B. Strategy used to humanized the 3 amino-acids G676R, F681Y, R684H in the murine *App* gene with localisation of the homology arms (green) used to target the locus with the loxP-Neo-LoxP selection cassette to isolate the recombinant ES clones. The cassette was removed before establishing the line. C. Sequence of the recombinant allele carrying the 3 mutations with changes in the amino-acid sequence. D. Strategy to introgress the App^{H} allele in the Ts66Yah and Dp(16)1Yey mouse line to generate the Ts68Yah and the Dp(16)15Yah mouse line. Blue box to show the breeding strategies to produce mutants for the analysis. E. The two closely related Ts66Yah and Ts68Yah lines were crossed with wt and $App^{H/H}$ to generate, in parallel, cohorts comprising the four genotypes used for the analysis.

Figure S2: Spatial learning in the Morris water maze for the Ts68Yah DS model
The two cohorts of mice at 3 and 9 months of ages were trained to learn where the platform was located during 6 days. We scored the latency to reach the platform, the distance travelled and estimated the swimming speed but we did not detect any phenotypes with statistically significant difference.

Figure S3: GDAPHEN analysis of the 48 behavioural variables analysed from the 4 genotype groups, WT, Ts66Yah, $App^{H2,}$ and Ts68Yah, highlighted that both 9- and 3-months-derived variables a contributes to the discrimination of the four genotypes. Notably 22 non-corelated behavioural variables (Supplementary table 3) were selected by the GDAPHEN analysis to discriminate the 4 genotypes, accounting for 33% of the total variance. Dimension 1 separates the non-humanised models (WT and Ts66Yah) from the humanised one (App^{H2} and Ts68Yah). Dimension 2 distinguishes trisomic genotypes (Ts66Yah and Ts68Yah) from euploid ones (WT and App^{H2}). At the top of the figure, individual data points are displayed in a 3D plot, annotated by genotype (left panel), then three 2D projections of the first three dimensions are shown (right). At the bottom, the contributions of the main behavioural variables to each 2D representation are illustrated in 3 panels, with each variable assigned a unique colour code.

Figure S4: Immunolocalisation of APP in the brain of Ts68Yah and control mice at different ages. A. intracellular APP immunolocalization using the 6E10 antibody on Ts68Yah brain cortical (Cx) or hippocampal section at 12 months of age with various fixation techniques.

B. Detection of Intracellular Aβ deposits with the 82E1 antibody in 3- and 20-month-old hippocampus of Ts68Yah mice compared to App^{H2} (Paraffin section 5µm)

Figure S5 Quantification of hippocampal full-length APP, APP C-terminal fragments in the Ts68Yah mouse model. Abundance of full-length APP (FL-APP) (A), C-terminal fragment alpha (CTF-α) (B), C-terminal fragment beta (CTF-β) (C) and the CTF-β/CTF-α ratio (D) were quantified in the hippocampus in wildtype (WT) (green), App^{H2} (brown), Ts66Yah (orange), and Ts68Yah (purple) mice at 3-months, 6-months and 12-months of age by western blot. Representative image of western blot of 3-months (E), 6-months (F), and 12-months (G) of age. FL-APP abundance (A) was significantly affected by amyloid-β humanisation at 3 months ($F(1,24) = 7.566$, $p = 0.011$) and by the presence of the minichromosome at 3 months ($F(1,24) = 11.809$, $p = 0.002$) and 12 months ($F(1,24) = 11.982$, $p = 0.002$) of age. CTF-α abundance (B) was significantly affected by amyloid-β humanisation at 3-months ($F(1,24) = 20.832$, $p < 0.001$) and by presence of minichromosome at 3-months ($F(1,24) = 12.952$, $p = 0.001$), 6-months $F(1,23) = 7.69$, $p = 0.011$) and 12 months ($F(1,24) = 28.8$, $p = < 0.001$) of age. CTF-β abundance (C) was significantly affected by amyloid-β humanisation at 6 months ($F(1,23) = 7.915$, $p = 0.01$) and 12 months ($F(1,24) = 22.908$, $p = < 0.001$) of age. The CTF-β/CTF-α ratio (D) was significantly affected by amyloid-β humanisation 3-months ($F(1,24) = 20.693$, $p < 0.001$), 6 months ($F(1,23) = 16.621$, $p < 0.001$), and 12 months ($F(1,24) = 34.502$, $p < 0.001$) of age, and by the presence of the minichromosome ($F(1,24) = 7.165$, $p = 0.013$) at 12 months of age. ANOVA with factors of amyloid-β humanisation, presence of minichromosome, and sex. Pairwise comparisons with Bonferroni correction for multiple comparisons, $p<0.05$ *, $p<0.01$ **, $p<0.001$ ***. Error bars SEM. Data points are biological replicates, female (red), male (blue), sex was included as a variable in the ANOVA (reported in supplementary Table 5). $App^{tm1Dbo/tm1Dbo}$ ($App^{-/-}$) samples were used as a negative control, and $App^{NL\text{-}G\text{-}F/NL\text{-}G\text{-}F}$ samples as a positive control.

Figure S6: Quantification of 82E1 staining in the Ts68Yah mouse model at 12-months of age. Percentage of cortex or hippocampus positive for 82E1 staining (N-terminal amyloid-β antibody in cortex (A) and hippocampus (B) of wildtype, Ts66Yah, App^{H2} and Ts68Yah mice at 12-months of age. Representative image of 82E1 (red) and DAPI (blue) staining of WT, App^{H2}, Ts66Yah and Ts68Yah animals, and an $App^{NL\text{-}G\text{-}F/NL\text{-}G\text{-}F}$ animal with positive 82E1 staining (C). ANOVA with factors of *App* humanisation, presence of minichromosome, and sex, found no difference between genotypes. Error bars SEM. Data-points are biological replicates. $App^{tm1Dbo/tm1Dbo}$(App-KO) samples were used as a negative control and $App^{NL\text{-}G\text{-}F/NL\text{-}G\text{-}F}$ samples were used as a positive control.

Figure S7 Quantification of hippocampal full-length APP, APP C-terminal fragments in the Dp(16)15Yah mouse model. Abundance of full-length APP (FL-APP) (A, E-G), C-terminal fragment alpha (CTF-α) (B, E-G), C-terminal fragment beta (CTF-β) (C, E-G), and the CTF-β/CTF-α ratio (D, E-G) were quantified in the hippocampus of wildtype (WT) (green), App^{H2} (brown), Dp(16)1Yey (yellow), and Dp(16)15Yah (teal) mice at 3 months, 6 months, and 12 months of age by western blot. Representative image of western blot of 3-month (E), 6-month (F), and 12-month-old (G). FL-APP abundance (A) was affected by amyloid-β humanisation at 6-months ($F(1,27) = 16.256$, $p < 0.001$); and the segmental duplication at 6-months ($F(1,27) = 13.711$, $p < 0.001$), 12-months ($F(1,26) = 6.238$, $p = 0.019$) of age, with a interaction between amyloid-β humanisation and segmental duplication observed at 6-months ($F(1,27) = 8.086$, $p = 0.008$) of age. CTF-α abundance (B) was affected by segmental duplication at 3-months ($F(1,24) = 11.328$, $p = 0.003$), 6-months $F(1,27) = 37.232$, $p < 0.001$), 12-months ($F(1,26) = 14.039$, $p < 0.001$), and amyloid-β humanisation at 6-months ($F(1,27) =$

28.632, $p < 0.001$) of age. CTF-β abundance (C) was significantly affected by amyloid-β humanisation at 3-months ($F(1,24) = 6.638$, $p = 0.017$), 6-months ($F(1,27) = 16.748$, $p < 0.001$), 12-months ($F(1,26) = 8.091$, $p = 0.009$), and the segmental duplication at 6-months ($F(1,27) = 4.209$, $p = 0.05$) of age. The CTF-β/CTF-α ratio (D) was affected by amyloid-β humanisation at 3-months ($F(1,24) = 9.48$, $p = 0.005$), 6-months ($F(1,27) = 82.725$, $p < 0.001$), 12-months ($F(1,26) = 63.136$, $p < 0.001$) and the segmental duplication of 6-months ($F(1,27) = 7.073$, $p = 0.013$), a interaction between amyloid-β humanisation and segmental duplication was observed at 6-months ($F(1,27) = 9.15$, $p = 0.005$) and 12-months ($F(1,26) = 9.532$, $p = 0.005$) of age. ANOVA with factors of amyloid-β humanisation, presence of segmental duplication, and sex was conducted for each time point. Pairwise comparisons with Bonferroni correction for multiple comparisons, p<0.05 *, p<0.01 **, p<0.001 ***. Error bars SEM. Data points are biological replicates, female (red), male (blue), sex was included as a variable in the ANOVA (reported in supplementary Table 5). $App^{tm1Dbo/tm1Dbo}$ ($App^{-/-}$) samples were used as a negative control, and $App^{NL\text{-}G\text{-}F/NL\text{-}G\text{-}F}$ samples as a positive control.

Figure S8. WB analysis of SOD1 in Ts66Yah and Ts68Yah Hippocampus. (A1) and (A2) Representative Western blots and densitometric evaluation of SOD1 total expression (B), in the hippocampus and cortex of mice at different ages, 3 months (WT n = 8, App^{H2} n = 8, Ts66Yah n = 8, Ts68Yah n = 8) and 9 months (WT n = 8, App^{H2} n = 8, Ts66 n = 8, Ts68 n = 8).

Figure S9. Heat-map results from 3-way ANOVA analysis taking into consideration the influence of genotypes (minichromosome and App^{H2}) and of sex in cortex and hippocampus from 3- and 9-month-old mice. The heatmap shows the significant influence of each factor and its interaction for all the proteins analyzed. The grey square indicates no significant values.

Fig S1

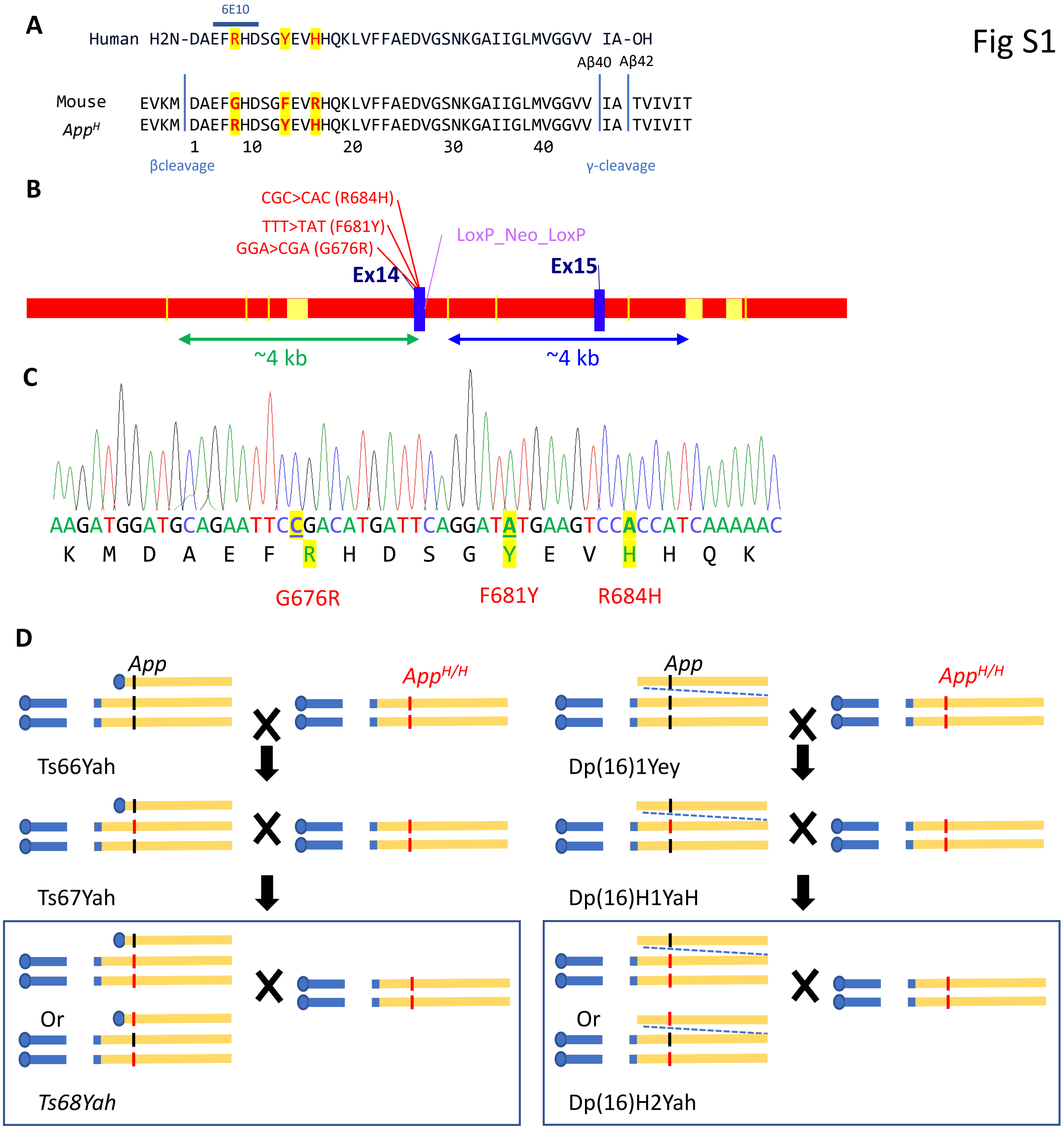

E

Ts66Yah X wt → Ts66Yah, Wt control

Ts68Yah X *App^H/H* → Ts68Yah, *App^H/H*

Fig S2

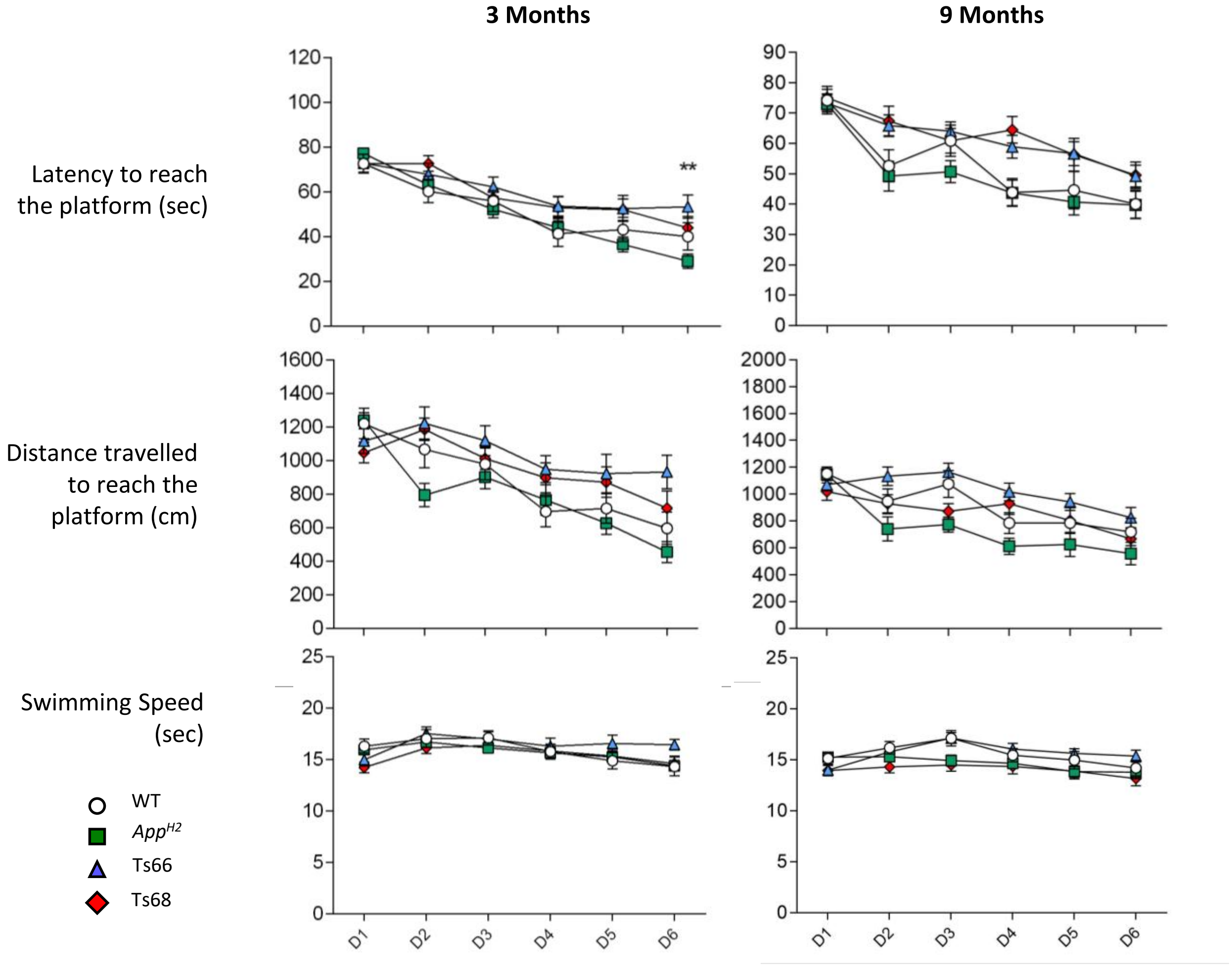

3 Months
9 Months
Latency to reach the platform (sec)
Distance travelled to reach the platform (cm)
Swimming Speed (sec)
WT
App^H2
Ts66
Ts68
**
D1
D2
D3
D4
D5
D6

Fig S3

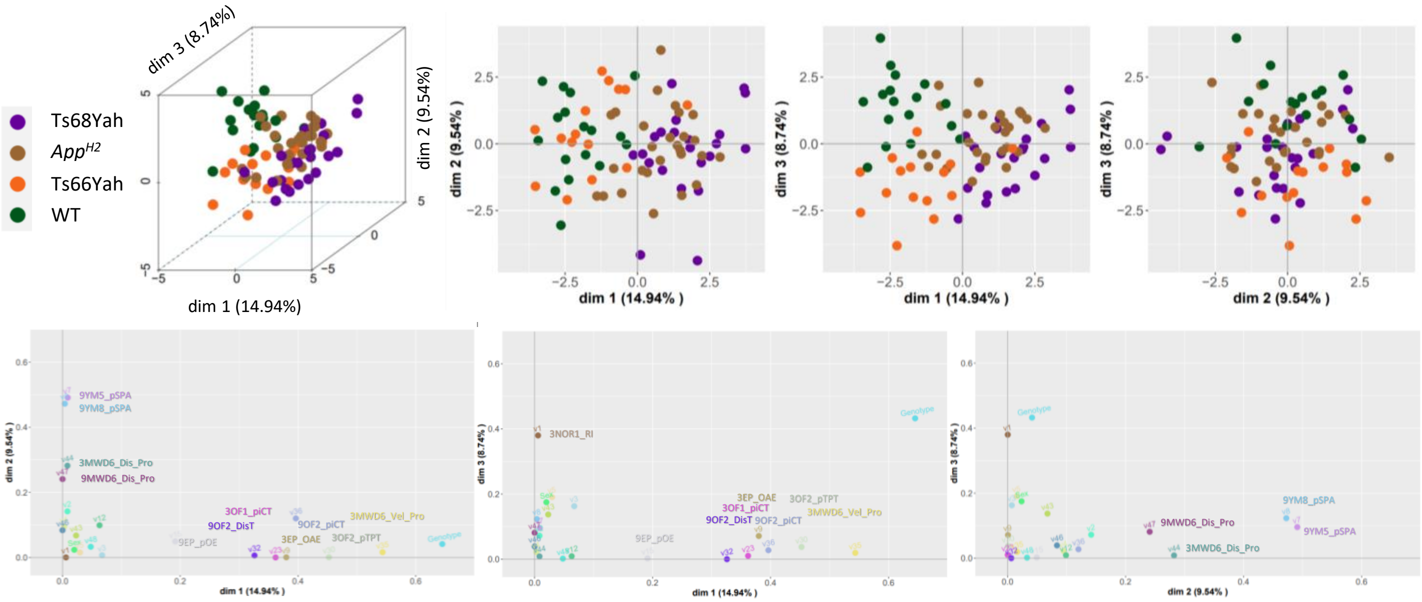
Ts68Yah
AppH2
Ts66Yah
WT
dim 1 (14.94%)
dim 2 (9.54%)
dim 3 (8.74%)
dim 1 (14.94%)
dim 2 (9.54%)
dim 3 (8.74%)
9YM5_pSPA
9YM8_pSPA
3MWD6_Dis_Pro
9MWD6_Dis_Pro
3OF1_piCT
9OF2_DisT
9OF2_piCT
3MWD6_Vel_Pro
9EP_pOE
3EP_OAE
3OF2_pTPT
Genotype
3NOR1_RI

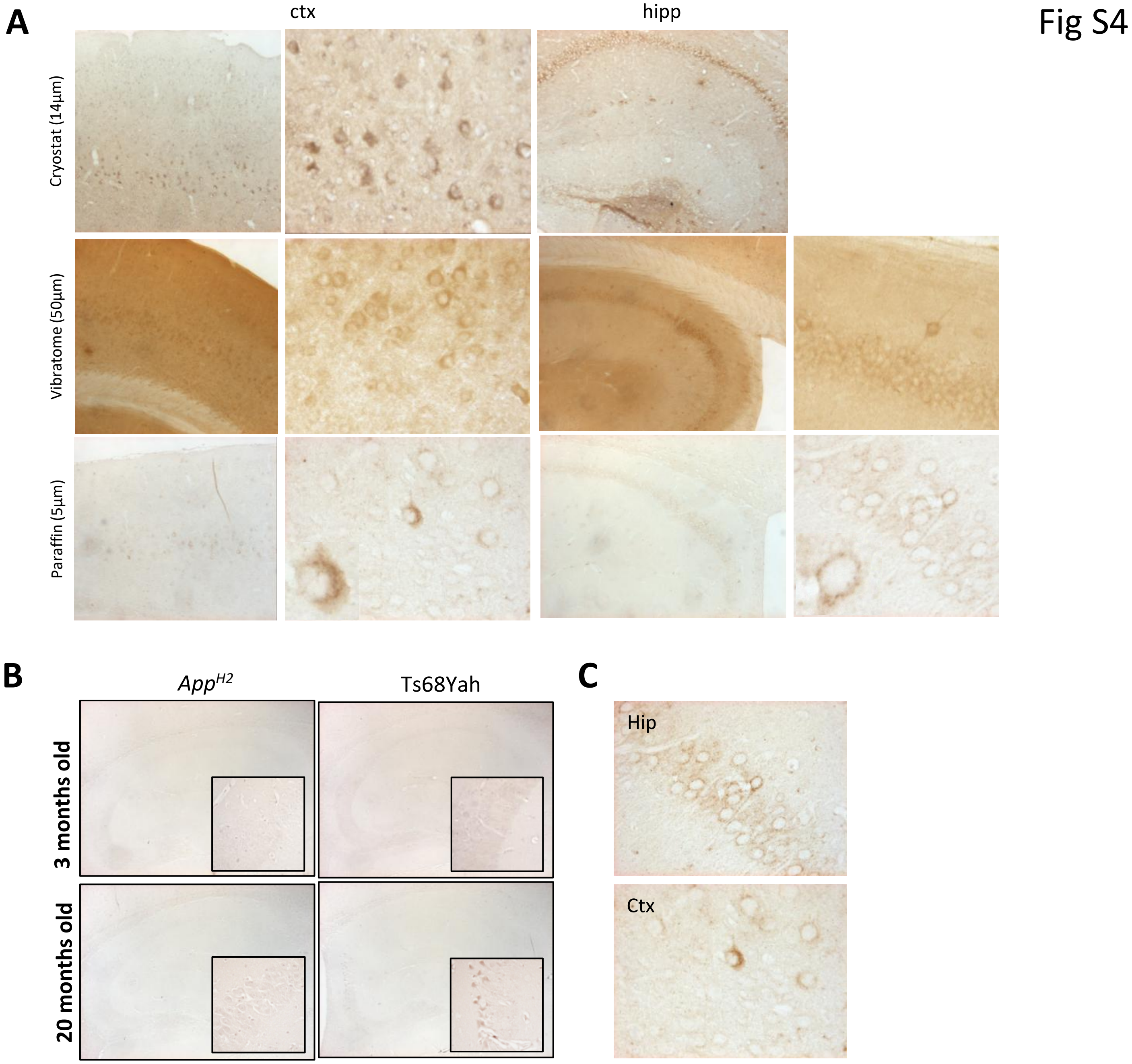
Fig S4
A
ctx
hipp
Cryostat (14µm)
Vibratome (50µm)
Paraffin (5µm)
B
App^H2
Ts68Yah
3 months old
20 months old
C
Hip
Ctx

Fig S5

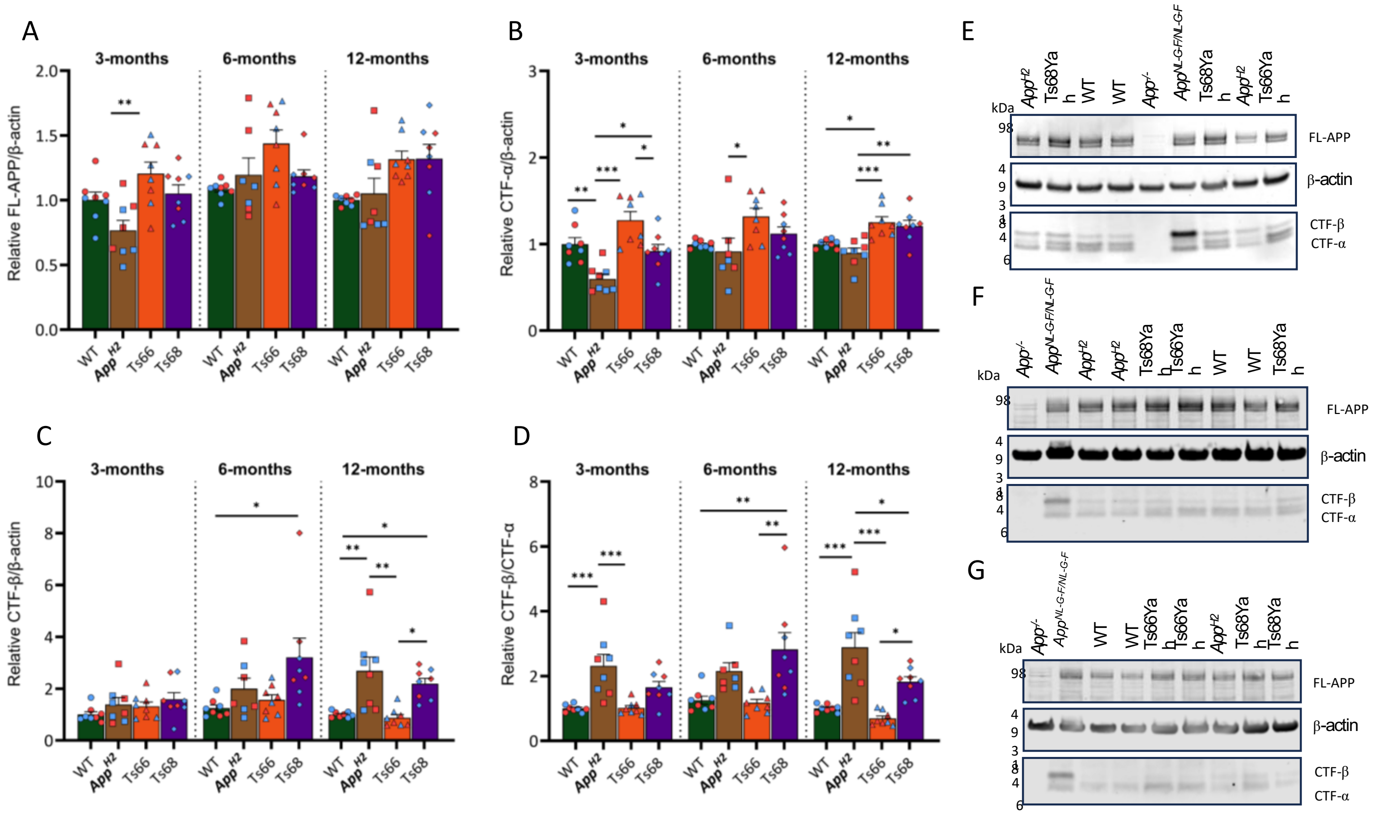

A
B
C
D
E
F
G
3-months
6-months
12-months
Relative FL-APP/β-actin
Relative CTF-α/β-actin
Relative CTF-β/β-actin
Relative CTF-β/CTF-α
WT
App H2
Ts66
Ts68
kDa
FL-APP
β-actin
CTF-β
CTF-α

Fig S6

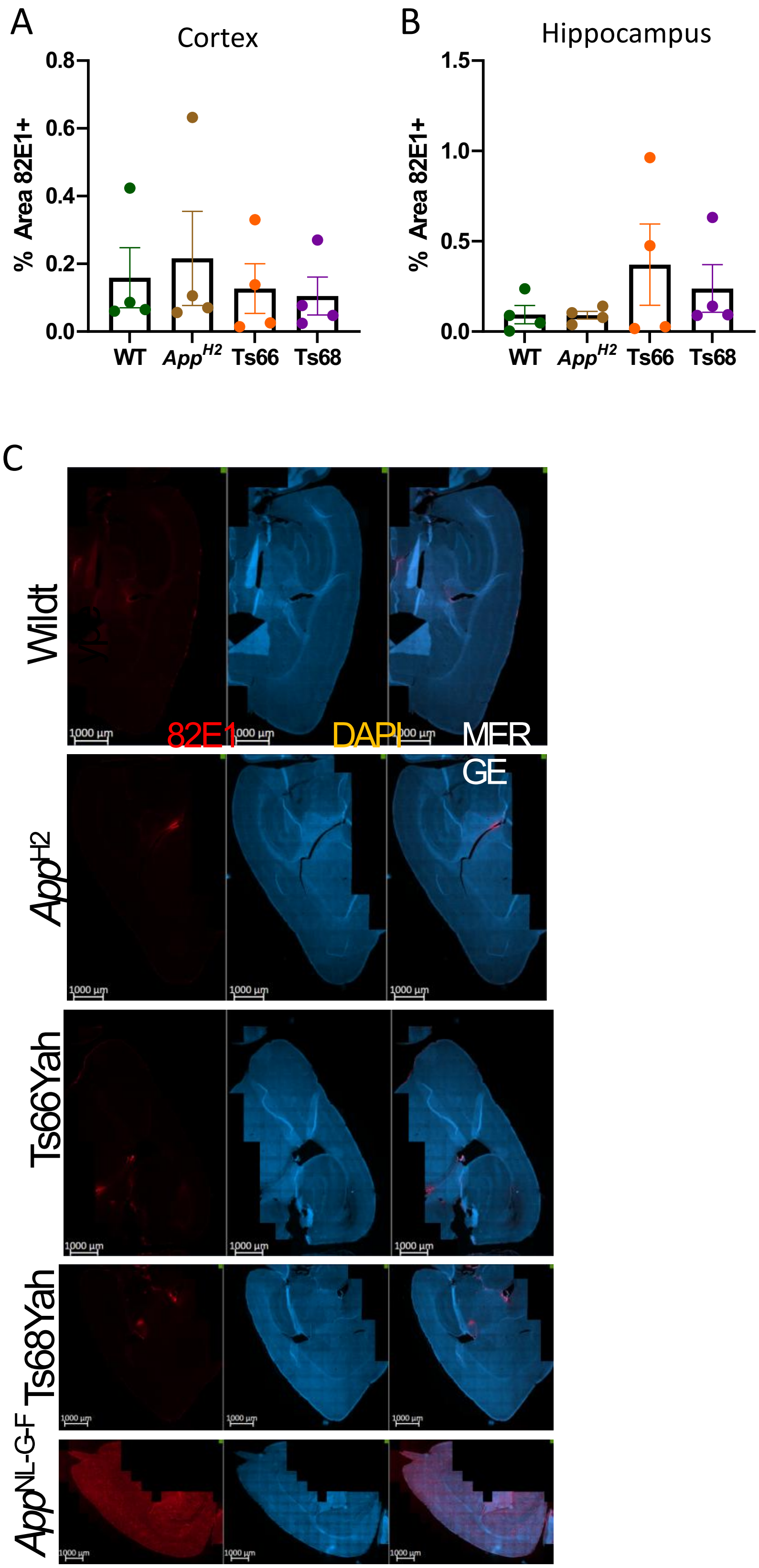
A
Cortex
% Area 82E1+
0.8
0.6
0.4
0.2
0.0
WT
App^{H2}
Ts66
Ts68
B
Hippocampus
% Area 82E1+
1.5
1.0
0.5
0.0
WT
App^{H2}
Ts66
Ts68
C
Wildt
82E1
DAPI
MER
GE
App^{H2}
Ts66Yah
Ts68Yah
App^{NL-G-F}
1000 µm

Fig S7

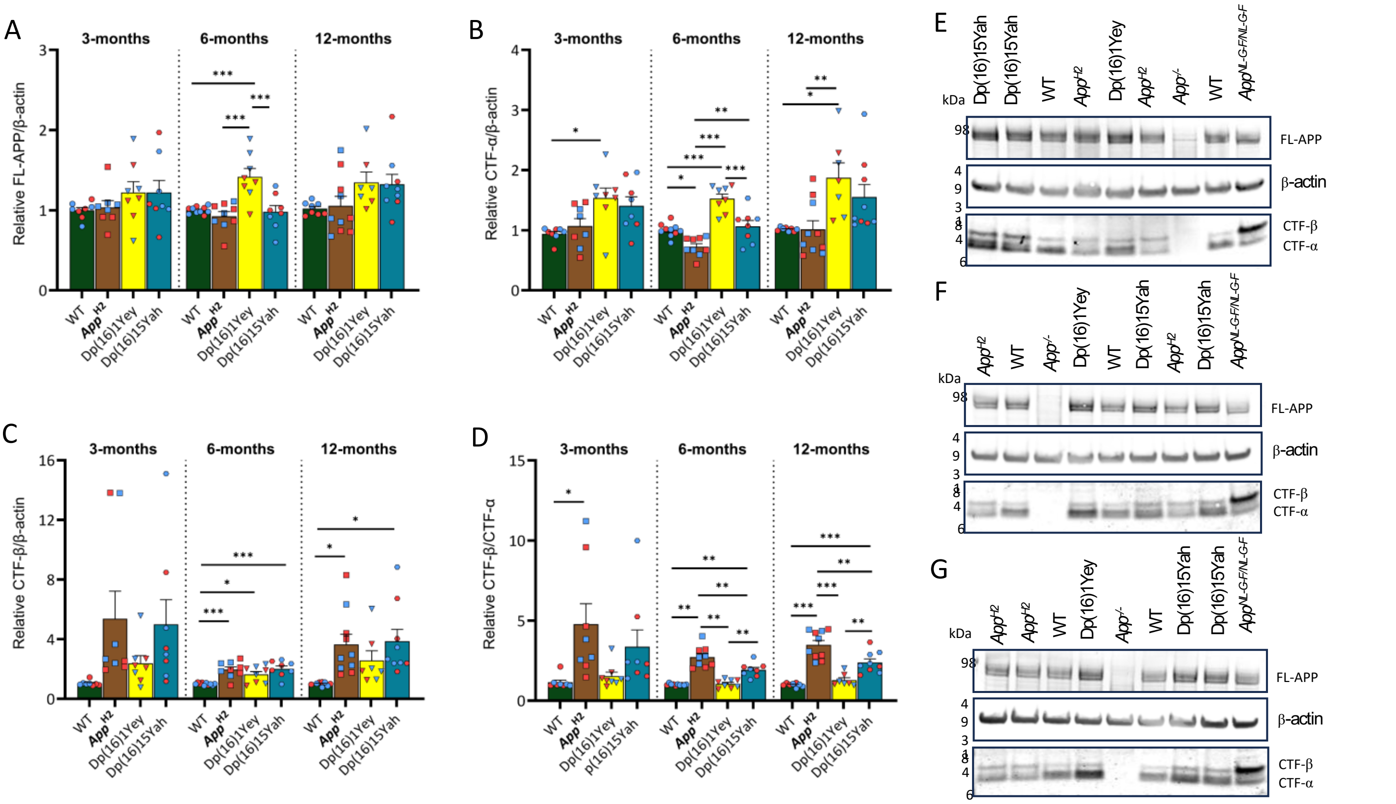

A
B
C
D
E
F
G
3-months
6-months
12-months
Relative FL-APP/β-actin
Relative CTF-α/β-actin
Relative CTF-β/β-actin
Relative CTF-β/CTF-α
WT
App H2
Dp(16)1Yey
Dp(16)15Yah
kDa
98
49
38
14
6
FL-APP
β-actin
CTF-β
CTF-α
App-/-
App NL-G-F/NL-G-F

Fig S8

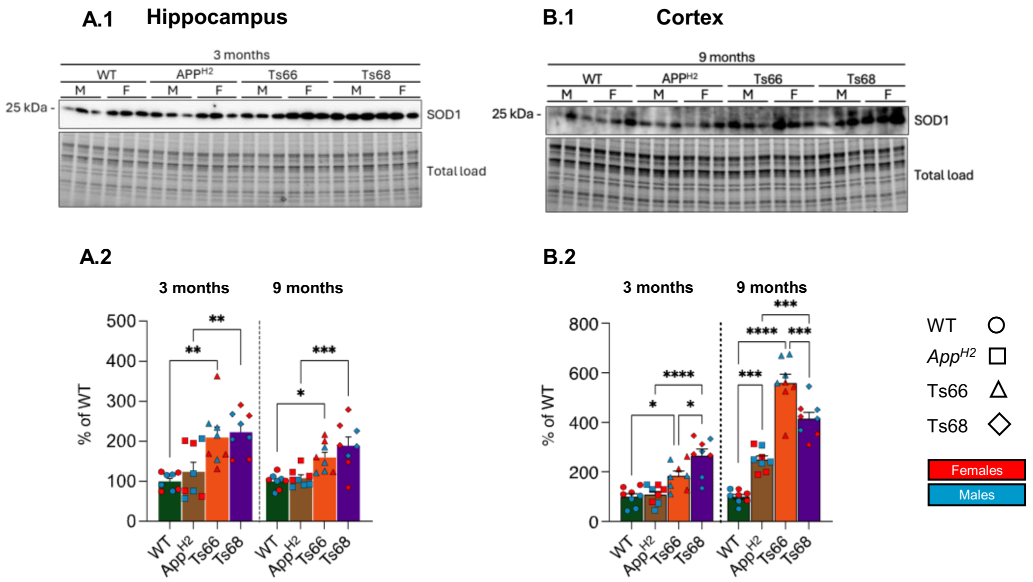

A.1
Hippocampus
3 months
WT
APPH2
Ts66
Ts68
M
F
25 kDa -
SOD1
Total load
B.1
Cortex
9 months
25 kDa -
SOD1
Total load
A.2
3 months
9 months
% of WT
500
400
300
200
100
0
WT
AppH2
Ts66
Ts68
B.2
800
600
400
200
0
WT
AppH2
Ts66
Ts68
Females
Males

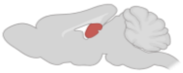

Hippocampus

3 months old mice

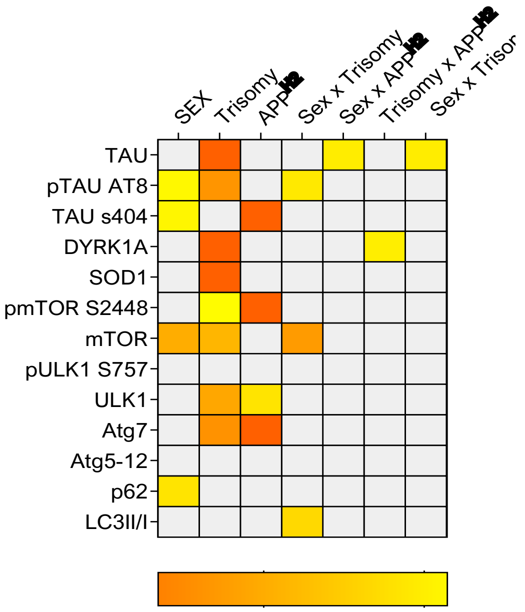

9 months old mice

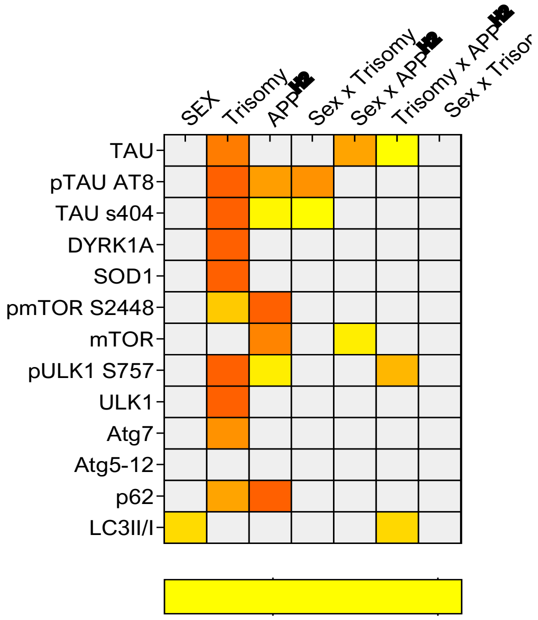

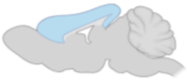

Cortex

3 months old mice

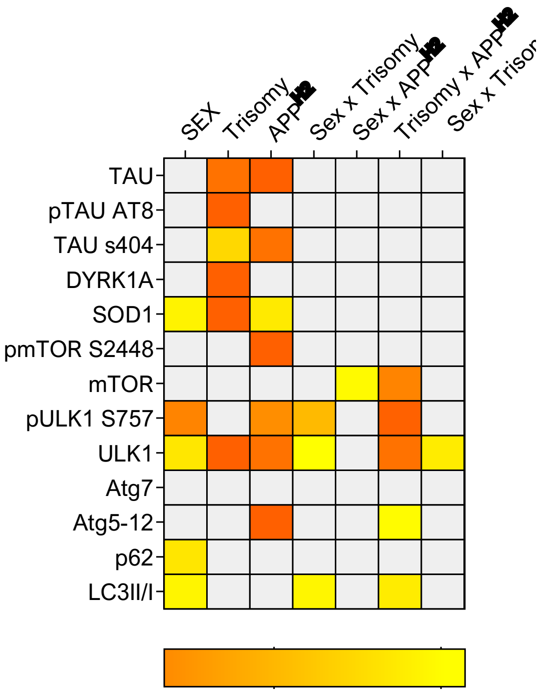

9 months old mice

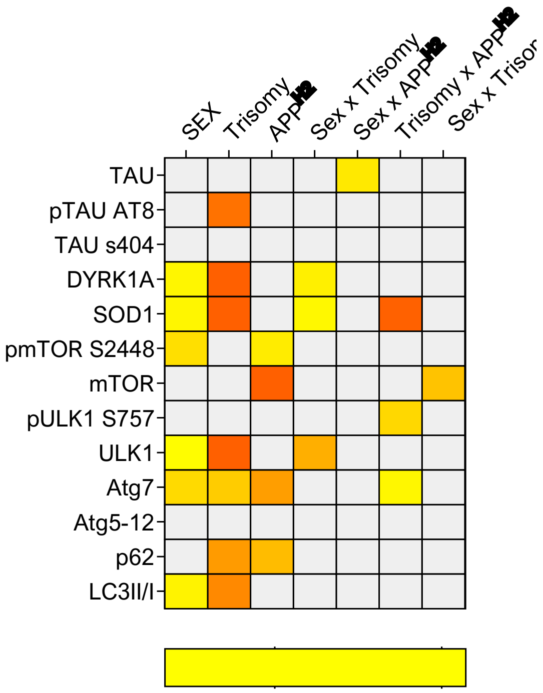

Supplement: Supplementary file 2 — Supporting Information: alz71498‐sup‐0002‐FiguresS1‐S9.pdf [file ALZ-22-e71498-s006.pdf]
