# Supplementary material for: Age‐related behavioral and molecular landmarks in new mouse models for studying Alzheimer's disease in Down syndrome
Source: Alzheimers Dement. 2026 May 21;22(5):e71498. doi: 10.1002/alz.71498 (PMC13240120; doi:10.1002/alz.71498)
Supplement: Supplementary file 3 — Supporting Information: alz71498‐sup‐0003‐TableS1.docx [file ALZ-22-e71498-s007.docx]

**Supplementary table 1: Mouse cohorts**

|  |  | | **n** | | | | | | | |
| --- | --- | --- | --- | --- | --- | --- | --- | --- | --- | --- |
|  |  | | **Female** | | | | **Male** | | | |
| **Figures** | | **Cohort (time points)** | **WT** | **Ts66Yah** | ***App^H2^*** | **Ts68Yah** | **WT** | **Ts66Yah** | ***App^H2^*** | **Ts68Yah** |
| 1 & 2 (S2, S3) | | B3 (3-months) | 11 | 9 | 14 | 14 | 9 | 8 | 16 | 22 |
| 1 & 2 (S2, S3) | | B9 (9-months) | 11 | 9 | 22 | 14 | 9 | 8 | 24 | 22 |
| 3 (S4) | | H3 (3-months) |  |  |  |  | 3 | 3 | 3 | 4 |
|  | | H12 (12-months) |  |  |  |  | 3 | 3 | 3 | 4 |
|  | | H20 (20-months) |  |  |  |  | 3 | 3 | 3 | 3 |
| 4 | | H9 (9-months) |  |  |  |  | 2 |  | 2 | 4 |
| 5 & 6 (S5, S6) | | A3 (3-months) | 4 | 4 | 4 | 4 | 4 | 4 | 4 | 4 |
| 5 & 6 (S5, S6) | | A6 (6-months) | 4 | 4 | 4 | 4 | 4 | 4 | 4 | 4 |
| 5 & 6 (S5, S6) | | A12 (12-months) | 4 | 4 | 4 | 4 | 4 | 4 | 4 | 4 |
| 7 & 8 | | M3 (3-months) | 4 | 4 | 4 | 4 | 4 | 4 | 4 | 4 |
| 7 & 8 | | M9 (9-months | 4 | 4 | 4 | 4 | 4 | 4 | 4 | 4 |
