# Supplementary material for: Age‐related behavioral and molecular landmarks in new mouse models for studying Alzheimer's disease in Down syndrome
Source: Alzheimers Dement. 2026 May 21;22(5):e71498. doi: 10.1002/alz.71498 (PMC13240120; doi:10.1002/alz.71498)
Supplement: Supplementary file 4 — Supporting Information: alz71498‐sup‐0004‐TableS2.docx [file ALZ-22-e71498-s001.docx]

**Supplementary table 2 : Primary antibodies**

| **Target** | **Host** | **Type** | **Provider** | **Catalogue** | **Dilution** |
| --- | --- | --- | --- | --- | --- |
| APP , CTFs (Y188) | Rabbit | Monoclonal | Abcam | ab32136 | 1 :10000 |
| APP, CTFs (A8717) | Rabbit | Polyclonal | MERCK | A8717 | 1 :10000 |
| β-actin | Mouse | Monoclonal | MERCK | A5441 | 1 :10000 |
| APP N-ter 22C11 | Mouse | Monoclonal | Thermo Fisher Scientific | 14-9749-80 | 1 :1000 |
| 6E10 reactive to aa 1-16 Aβ and to APP | Mouse | Monoclonal | Biolegend | 803001 | 1 :1000 |
| 82E1 Amyloid β N-terminal specific | Mouse | Monoclonal | IBL | 10323 | 1 :1000 |
| Tau total | Rabbit | Polyclonal | Byorbit | orb-46243 | 1 :500 |
| phosphoTau (Ser202, Thr205) AT8 | Mouse | Monoclonal | Thermo Fisher Scientific | MN1020 | 1 :1000 |
| phosphoTau (Ser404) | Rabbit | Monoclonal | Abcam | ab92676 | 1 :1000 |
| Atg7 | Rabbit | Monoclonal | Cell signaling Technology | D12B11 | 1 :1000 |
| Atg5-Atg12 | Recombinant | Monoclonal | Invitrogen | #702433 | 1 :1000 |
| SOD1 (aa120-146) | Mouse | Monoclonal | Santa Cruz Biotechnology | sc-271014 | 1 :500 |
| phospho-mTOR(Ser2448) | Rabbit | Monoclonal | Cell signaling Technology | #5536S | 1 :1000 |
| mTOR | Mouse | Monoclonal | Biolegend | 6H9B10 | 1 :1000 |
| DYRK1A D30C10 | Rabbit | Monoclonal | Cell signaling Technology | #8765 | 1 :1000 |
| SQSTM1/p62 | Rabbit | Polyclonal | Genetex | GTX100685 | 1 :1000 |
| ULK 1 | Rabbit | Monoclonal | Cell signaling Technology | #8054S | 1 :1000 |
| phospho-ULK1 (ser757) | Rabbit | Polyclonal | Invitrogen | PA5-105130 | 1 :1000 |
| LC3B | Rabbit | Polyclonal | Novus Biologicals | NB-1002220 | 1 :1000 |
