# Supplementary material for: Age‐related behavioral and molecular landmarks in new mouse models for studying Alzheimer's disease in Down syndrome
Source: Alzheimers Dement. 2026 May 21;22(5):e71498. doi: 10.1002/alz.71498 (PMC13240120; doi:10.1002/alz.71498)
Supplement: Supplementary file 5 — Supporting Information: alz71498‐sup‐0005‐TableS3.docx [file ALZ-22-e71498-s002.docx]

**Supplementary table 3: List of the variables used in the GDAPHEN analysis**

| **Nb Variable** | **Variable ShortCut** | **Variable Extended name** |
| --- | --- | --- |
|  | Sex | Sex |
|  | Genotype | Genotype |
| V1 | 3NOR1_RI | Recognition index from the novel object recognition test after 1h of retention in the 3-month-old cohort |
| V2 | 3NOR24_RI | Recognition index from the novel object recognition test after 24h of retention in the 3-month-old cohort |
| V3 | 9NOR1_RI | Recognition index from the novel object recognition test after 1h of retention in the 9-month-old cohort |
| V4 | 9NOR24_RI | Recognition index from the novel object recognition test after 24h of retention in the 9-month-old cohort |
| V5 | 3YM5_pSPA | percentage of spontaneous alternation after 5min in the Y maze test for the 3-month-old cohort |
| V6 | 3YM8_pSPA | percentage of spontaneous alternation after 8min in the Y maze test for the 3-month-old cohort |
| V7 | 9YM5_pSPA | percentage of spontaneous alternation after 5min in the Y maze test for the 9-month-old cohort |
| V8 | 9YM8_pSPA | percentage of spontaneous alternation after 8min in the Y maze test for the 9-month-old cohort |
| V9 | 3EP_OAE | nb of open arm entries in the elevated plus maze for the 3-month-old cohort |
| V10 | 3EP_pOE | Percentage of open arm entries in the elevated plus maze for the 3-month-old cohort |
| V11 | 3EP_OT | Time in open arms in the elevated plus maze for the 3-month-old cohort |
| V12 | 3EP_pOT | Percentage of time in open arms in the elevated plus maze for the 3-month-old cohort |
| V13 | 3EP_pOT_0+C | Percentage of time in open+close arms in the elevated plus maze for the 3-month-old cohort |
| V14 | 9EP_OAE | nb of open arm entries in the elevated plus maze for the 9-month-old cohort |
| V15 | 9EP_pOE | Percentage of open arm entries in the elevated plus maze for the 9-month-old cohort |
| V16 | 9EP_OT | Time in open arms in the elevated plus maze for the 9-month-old cohort |
| V17 | 9EP_pOT | Percentage of time in open arms in the elevated plus maze for the 9-month-old cohort |
| V18 | 9EP_pOT_0+C | Percentage of time in open+close arms in the elevated plus maze for the 9-month-old cohort |
| V19 | 3OF1_DisT | Distance travelled in the open field 1 in the 3-month-old cohort |
| V20 | 3OF2_DisT | Distance travelled in the open field 2 in the 3-month-old cohort |
| V21 | 3OF1_TiCT | Time in Centre in the open field 1 in the 3-month-old cohort |
| V22 | 3OF2_TiCT | Time in Centre in the open field 2 in the 3-month-old cohort |
| V23 | 3OF1_piCT | Percentage of time spent in the centre of the open field 1 in the 3-month-old cohort |
| V24 | 3OF2_piCT | Percentage of time spent in the centre of the open field 2 in the 3-month-old cohort |
| V25 | 3OF1_VelT | Velocity in the open field 1 in the 3-month-old cohort |
| V26 | 3OF2_VelT | Velocity in the open field 2 in the 3-month-old cohort |
| V27 | 3OF1_PerT | Time at the periphery in the open field 1 in the 3-month-old cohort |
| V28 | 3OF2_PerT | Time at the periphery in the open field 2 in the 3-month-old cohort |
| V29 | 3OF1_pTPT | Percentage of Time at the periphery in the open field 1 in the 3-month-old cohort |
| V30 | 3OF2_pTPT | Percentage of Time at the periphery in the open field 2 in the 3-month-old cohort |
| V31 | 9OF1_DisT | Distance travelled in the open field 1 in the 9-month-old cohort |
| V32 | 9OF2_DisT | Distance travelled in the open field 2 in the 9-month-old cohort |
| V33 | 9OF1_TiCT | Time in Centre in the open field 1 in the 9-month-old cohort |
| V34 | 9OF2_TiCT | Time in Centre in the open field 2 in the 9-month-old cohort |
| V35 | 9OF1_piCT | Percentage of time spent in the centre of the open field 1 in the 9-month-old cohort |
| V36 | 9OF2_piCT | Percentage of time spent in the centre of the open field 2 in the 9-month-old cohort |
| V37 | 9OF1_VelT | Velocity in the open field 1 in the 9-month-old cohort |
| V38 | 9OF2_VelT | Velocity in the open field 2 in the 9-month-old cohort |
| V39 | 9OF1_PerT | Time at the periphery in the open field 1 in the 9-month-old cohort |
| V40 | 9OF2_PerT | Time at the periphery in the open field 2 in the 9-month-old cohort |
| V41 | 9OF1_pTPT | Percentage of Time at the periphery in the open field 1 in the 9-month-old cohort |
| V42 | 9OF2_pTPT | Percentage of Time at the periphery in the open field 2 in the 9-month-old cohort |
| V43 | 3MWPT_SOplat_Lat | Percentage of time in the target quadrant in the probet test of the Morris water maze in the 3-month-old cohort |
| V44 | 3MWD6_Dis_Pro | Distance travelled to find the platform tin the probet test of the Morris water maze in the 3-month-old cohort |
| V45 | 3MWD6_Vel_Pro | Velocity to find the platform tin the probet test of the Morris water maze in the 3-month-old cohort |
| V46 | 9MWPT_SOplat_Lat | Percentage of time in the target quadrant in the probet test of the Morris water maze in the 9-month-old cohort |
| V47 | 9MWD6_Dis_Pro | Distance travelled to find the platform tin the probet test of the Morris water maze in the 9-month-old cohort |
| V48 | 9MWD6_Vel_Pro | Velocity to find the platform tin the probet test of the Morris water maze in the 9-month-old cohort |
