# Supplementary material for: Age‐related behavioral and molecular landmarks in new mouse models for studying Alzheimer's disease in Down syndrome
Source: Alzheimers Dement. 2026 May 21;22(5):e71498. doi: 10.1002/alz.71498 (PMC13240120; doi:10.1002/alz.71498)
Supplement: Supplementary file 6 — Supporting Information: alz71498‐sup‐0006‐TableS4.docx [file ALZ-22-e71498-s003.docx]

Supplementary table 5

ANOVA summary statistics for the effect of sex and the interaction of sex with other variables in cortical samples, genotype effects presented in Figures 5 and 6 as indicated.

| **Figure** | **Age (months)** | **Analyte** | **Variable/Interaction** | **ANOVA and Bonferroni Post hoc**  **p<0.05** |
| --- | --- | --- | --- | --- |
| 5A | 3 | Cortical FL-APP | Sex | F(1,24)=1.453 p=0.24 |
| 5A | 3 | Cortical FL-APP | Sex*Humanisation | F(1,24)=1.932 p=0.177 |
| 5A | 3 | Cortical FL-APP | Sex*Minichromosome | F(1,24)=1.945 p=0.176 |
| 5A | 3 | Cortical FL-APP | Sex*Humanisation*Minichromosome | F(1,24)=0.715 p=0.406 |
| 5A | 6 | Cortical FL-APP | Sex | F(1,23)=0.231 p=0.636 |
| 5A | 6 | Cortical FL-APP | Sex*Humanisation | F(1,23)=0.766 p=0.39 |
| 5A | 6 | Cortical FL-APP | Sex*Minichromosome | F(1,23)=6.952 p=0.015  Posthoc  Female (WTvTs66 p=0.02; WTvTs68 p=0.012; App^H2^vTs66 p=0.01; App^H2^ vTs68 p=0.04)  Male (App^H2^vTs66 p=0.045) |
| 5A | 6 | Cortical FL-APP | Sex*Humanisation*Minichromosome | F(1,23)=0.522 p=0.477 |
| 5A | 12 | Cortical FL-APP | Sex | F(1,24)=0.244 p=0.626 |
| 5A | 12 | Cortical FL-APP | Sex*Humanisation | F(1,24)=1.7 p=0.205 |
| 5A | 12 | Cortical FL-APP | Sex*Minichromosome | F(1,24)=2.395 p=0.135 |
| 5A | 12 | Cortical FL-APP | Sex*Humanisation*Minichromosome | F(1,24)=0.142 p=0.71 |
| 5B | 3 | Cortical CTF-α | Sex | F(1,24)=0.092 p=0.765 |
| 5B | 3 | Cortical CTF-α | Sex*Humanisation | F(1,24)=1.716 p=0.203 |
| 5B | 3 | Cortical CTF-α | Sex*Minichromosome | F(1,24)=0.21 p=0.651 |
| 5B | 3 | Cortical CTF-α | Sex*Humanisation*Minichromosome | F(1,24)=0.058 p=0.821 |
| 5B | 6 | Cortical CTF-α | Sex | F(1,23)=0.049 p=0.828 |
| 5B | 6 | Cortical CTF-α | Sex*Humanisation | F(1,23)=1.473 p=0.237 |
| 5B | 6 | Cortical CTF-α | Sex*Minichromosome | F(1,23)=0.95 p=0.34 |
| 5B | 6 | Cortical CTF-α | Sex*Humanisation*Minichromosome | F(1,23)=4.537 p=0.044  Posthoc  Female (WTvTs66 p=0.004; App^H2^vTs66 p=0.007; Ts66 vTs68 p=0.004)  Male not significant |
| 5B | 12 | Cortical CTF-α | Sex | F(1,24)=0.197 p=0.661 |
| 5B | 12 | Cortical CTF-α | Sex*Humanisation | F(1,24)=3.096 p=0.091 |
| 5B | 12 | Cortical CTF-α | Sex*Minichromosome | F(1,24)=0.26 p=0.615 |
| 5B | 12 | Cortical CTF-α | Sex*Humanisation*Minichromosome | F(1,24)=0.026 p=0.874 |
| 5C | 3 | Cortical CTF-β | Sex | F(1,24)=0.824 p=0.373 |
| 5C | 3 | Cortical CTF-β | Sex*Humanisation | F(1,24)=1.748 p=0.199 |
| 5C | 3 | Cortical CTF-β | Sex*Minichromosome | F(1,24)=0.046 p=0.831 |
| 5C | 3 | Cortical CTF-β | Sex*Humanisation*Minichromosome | F(1,24)>0.001 p=0.989 |
| 5C | 6 | Cortical CTF-β | Sex | F(1,23)=0.68 p=0.418 |
| 5C | 6 | Cortical CTF-β | Sex*Humanisation | F(1,23)=1.846 p=0.187 |
| 5C | 6 | Cortical CTF-β | Sex*Minichromosome | F(1,23)=5.05 p=0.035  Posthoc  Female (WTvTs68 p=0.018)  Male (WTvApp^H2^ p<0.001; WTvTs68 p=0.001; App^H2^vTs66 p<0.001; Ts66vTs68 p=0.002) |
| 5C | 6 | Cortical CTF-β | Sex*Humanisation*Minichromosome | F(1,23)=0.039 p=0.845 |
| 5C | 12 | Cortical CTF-β | Sex | F(1,24)=0.044 p=0.836 |
| 5C | 12 | Cortical CTF-β | Sex*Humanisation | F(1,24)=0.075 p=0.787 |
| 5C | 12 | Cortical CTF-β | Sex*Minichromosome | F(1,24)=2.878 p=0.103 |
| 5C | 12 | Cortical CTF-β | Sex*Humanisation*Minichromosome | F(1,24)=1.661 p=0.21 |
| 5D | 3 | Cortical CTF-β/CTF-α | Sex | F(1,24)=0.16 p=0.693 |
| 5D | 3 | Cortical CTF-β/CTF-α | Sex*Humanisation | F(1,24)=0.049 p=0.827 |
| 5D | 3 | Cortical CTF-β/CTF-α | Sex*Minichromosome | F(1,24)=0.259 p=0.615 |
| 5D | 3 | Cortical CTF-β/CTF-α | Sex*Humanisation*Minichromosome | F(1,24)=0.004 p=0.953 |
| 5D | 6 | Cortical CTF-β/CTF-α | Sex | F(1,23)=0.442 p=0.513 |
| 5D | 6 | Cortical CTF-β/CTF-α | Sex*Humanisation | F(1,23)=0.67 p=0.421 |
| 5D | 6 | Cortical CTF-β/CTF-α | Sex*Minichromosome | F(1,23)=3.633 p=0.069 |
| 5D | 6 | Cortical CTF-β/CTF-α | Sex*Humanisation*Minichromosome | F(1,23)=0.344 p=0.563 |
| 5D | 12 | Cortical CTF-β/CTF-α | Sex | F(1,24)=0.013 p=0.911 |
| 5D | 12 | Cortical CTF-β/CTF-α | Sex*Humanisation | F(1,24)=0.002 p=0.969 |
| 5D | 12 | Cortical CTF-β/CTF-α | Sex*Minichromosome | F(1,24)=4.172 p=0.052 |
| 5D | 12 | Cortical CTF-β/CTF-α | Sex*Humanisation*Minichromosome | F(1,24)=2.445 p=0.131 |
|  |  |  |  |  |
| 5F | 3 | Cortical FL-APP | Sex | F(1,24)<0.001 p=0.996 |
| 5F | 3 | Cortical FL-APP | Sex*Humanisation | F(1,24)=0.232 p=0.634 |
| 5F | 3 | Cortical FL-APP | Sex*Duplication | F(1,24)=0.058 p=0.811 |
| 5F | 3 | Cortical FL-APP | Sex*Humanisation*Duplication | F(1,24)=0.111 p=0.742 |
| 5F | 6 | Cortical FL-APP | Sex | F(1,29)=2.581 p=0.119 |
| 5F | 6 | Cortical FL-APP | Sex*Humanisation | F(1,29)=0.759 p=0.391 |
| 5F | 6 | Cortical FL-APP | Sex*Duplication | F(1,29)=2.168 p=0.152 |
| 5F | 6 | Cortical FL-APP | Sex*Humanisation*Duplication | F(1,29)=4.044 p=0.054 |
| 5F | 12 | Cortical FL-APP | Sex | F(1,27)=0.133 p=0.718 |
| 5F | 12 | Cortical FL-APP | Sex*Humanisation | F(1,27)=1.423 p=0.243 |
| 5F | 12 | Cortical FL-APP | Sex*Duplication | F(1,27)=0.053 p=0.819 |
| 5F | 12 | Cortical FL-APP | Sex*Humanisation*Duplication | F(1,27)=0.337 p=0.566 |
| 5G | 3 | Cortical CTF-α | Sex | F(1,24)=0.021 p=0.885 |
| 5G | 3 | Cortical CTF-α | Sex*Humanisation | F(1,24)=1.517 p=0.23 |
| 5G | 3 | Cortical CTF-α | Sex*Duplication | F(1,24)=1.693 p=0.206 |
| 5G | 3 | Cortical CTF-α | Sex*Humanisation*Duplication | F(1,23)=0.013 p=0.909 |
| 5G | 6 | Cortical CTF-α | Sex | F(1,29)=2.633 p=0.116 |
| 5G | 6 | Cortical CTF-α | Sex*Humanisation | F(1,29)=1.085 p=0.306 |
| 5G | 6 | Cortical CTF-α | Sex*Duplication | F(1,29)=1.948 p=0.173 |
| 5G | 6 | Cortical CTF-α | Sex*Humanisation*Duplication | F(1,29)=4.172 p=0.050 |
| 5G | 12 | Cortical CTF-α | Sex | F(1,27)=0.392 p=0.537 |
| 5G | 12 | Cortical CTF-α | Sex*Humanisation | F(1,27)=0.419 p=0.523 |
| 5G | 12 | Cortical CTF-α | Sex*Duplication | F(1,27)=0.253 p=0.619 |
| 5G | 12 | Cortical CTF-α | Sex*Humanisation*Duplication | F(1,27)=0.02 p=0.887 |
| 5H | 3 | Cortical CTF-β | Sex | F(1,24)=0.136 p=0.715 |
| 5H | 3 | Cortical CTF-β | Sex*Humanisation | F(1,24)=0.871 p=0.36 |
| 5H | 3 | Cortical CTF-β | Sex*Duplication | F(1,24)=0.405 p=0.531 |
| 5H | 3 | Cortical CTF-β | Sex*Humanisation*Duplication | F(1,24)=0.262 p=0.614 |
| 5H | 6 | Cortical CTF-β | Sex | F(1,29)=0.085 p=0.773 |
| 5H | 6 | Cortical CTF-β | Sex*Humanisation | F(1,29)=0.209 p=0.651 |
| 5H | 6 | Cortical CTF-β | Sex*Duplication | F(1,29)=0.391 p=0.537 |
| 5H | 6 | Cortical CTF-β | Sex*Humanisation*Duplication | F(1,29)=0.907 p=0.349 |
| 5H | 12 | Cortical CTF-β | Sex | F(1,27)=0.041 p=0.841 |
| 5H | 12 | Cortical CTF-β | Sex*Humanisation | F(1,27)=0.092 p=0.764 |
| 5H | 12 | Cortical CTF-β | Sex*Duplication | F(1,27)=0.647 p=0.428 |
| 5H | 12 | Cortical CTF-β | Sex*Humanisation*Duplication | F(1,27)=0.005 p=0.944 |
| 5I | 3 | Cortical CTF-β/CTF-α | Sex | F(1,24)=0.826 p=0.373 |
| 5I | 3 | Cortical CTF-β/CTF-α | Sex*Humanisation | F(1,24)<0.001 p=0.999 |
| 5I | 3 | Cortical CTF-β/CTF-α | Sex*Duplication | F(1,24)=0.083 p=0.775 |
| 5I | 3 | Cortical CTF-β/CTF-α | Sex*Humanisation*Duplication | F(1,24)=0.689 p=0.415 |
| 5I | 6 | Cortical CTF-β/CTF-α | Sex | F(1,29)=1.621 p=0.213 |
| 5I | 6 | Cortical CTF-β/CTF-α | Sex*Humanisation | F(1,29)=0.168 p=0.685 |
| 5I | 6 | Cortical CTF-β/CTF-α | Sex*Duplication | F(1,29)=0.49 p=0.49 |
| 5I | 6 | Cortical CTF-β/CTF-α | Sex*Humanisation*Duplication | F(1,29)=0.768 p=0.388 |
| 5I | 12 | Cortical CTF-β/CTF-α | Sex | F(1,27)=0.014 p=0.908 |
| 5I | 12 | Cortical CTF-β/CTF-α | Sex*Humanisation | F(1,27)=0.083 p=0.775 |
| 5I | 12 | Cortical CTF-β/CTF-α | Sex*Duplication | F(1,27)=0.318 p=0.577 |
| 5I | 12 | Cortical CTF-β/CTF-α | Sex*Humanisation*Duplication | F(1,27)=0.077 p=0.783 |
|  |  |  |  |  |
| 6A | 3 | Tris sol. amyloid-β_40_ | Sex | F(1,12)=3.949 p=0.07 |
| 6A | 3 | Tris sol. amyloid-β_40_ | Sex*Minichromosome | F(1,12)=0.309 p=0.589 |
| 6A | 6 | Tris sol. amyloid-β_40_ | Sex | F(1,11)=16.538 p=0.002 |
| 6A | 6 | Tris sol. amyloid-β_40_ | Sex*Minichromosome | F(1,11)=2.655 p=0.131 |
| 6A | 12 | Tris sol. amyloid-β_40_ | Sex | F(1,12)<0.001 p=0.999 |
| 6A | 12 | Tris sol. amyloid-β_40_ | Sex*Minichromosome | F(1,12)=1.018 p=0.333 |
| 6B | 3 | 1% Triton X100 sol. amyloid-β_40_ | Sex | F(1,12)=0.022 p=0.884 |
| 6B | 3 | 1% Triton X100 sol. amyloid-β_40_ | Sex*Minichromosome | F(1,12)=0.274 p=0.61 |
| 6B | 6 | 1% Triton X100 sol. amyloid-β_40_ | Sex | F(1,11)=22.781 p<0.001 |
| 6B | 6 | 1% Triton X100 sol. amyloid-β_40_ | Sex*Minichromosome | F(1,11)=13.062 p=0.004  Male *App^H2^*vTs68 not significant  Female *App^H2^*vTs68 F(1,5)=52.436, p=0.001 |
| 6B | 12 | 1% Triton X100 sol. amyloid-β_40_ | Sex | F(1,11)=0.739 p=0.418 |
| 6B | 12 | 1% Triton X100 sol. amyloid-β_40_ | Sex*Minichromosome | F(1,11)=2.583 p=0.152 |
| 6C | 3 | 5M GndHcl sol. amyloid-β_40_ | Sex | F(1,12)=1.177 p=0.299 |
| 6C | 3 | 5M GndHcl sol. amyloid-β_40_ | Sex*Minichromosome | F(1,12)=0.68 p=0.426 |
| 6C | 6 | 5M GndHcl sol. amyloid-β_40_ | Sex | F(1,11)=3.197 p=0.101 |
| 6C | 6 | 5M GndHcl sol. amyloid-β_40_ | Sex*Minichromosome | F(1,11)=0.148 p=0.708 |
| 6C | 12 | 5M GndHcl sol. amyloid-β_40_ | Sex | F(1,12)=3.419 p=0.089 |
| 6C | 12 | 5M GndHcl sol. amyloid-β_40_ | Sex*Minichromosome | F(1,12)=1.62 p=0.227 |
| 6D | 3 | Tris sol. amyloid-β_42_ | Sex | F(1,12)=4.331 p=0.06 |
| 6D | 3 | Tris sol. amyloid-β_42_ | Sex*Minichromosome | F(1,12)=0.006 p=0.941 |
| 6D | 6 | Tris sol. amyloid-β_42_ | Sex | F(1,11)=7.539 p=0.019 |
| 6D | 6 | Tris sol. amyloid-β_42_ | Sex*Minichromosome | F(1,11)=1.104 p=0.316 |
| 6D | 12 | Tris sol. amyloid-β_42_ | Sex | F(1,12)=1.39 p=0.261 |
| 6D | 12 | Tris sol. amyloid-β_42_ | Sex*Minichromosome | F(1,12)=0.742 p=0.406 |
| 6E | 3 | 1% Triton X100 sol. amyloid-β_42_ | Sex | F(1,12)=0.308 p=0.589 |
| 6E | 3 | 1% Triton X100 sol. amyloid-β_42_ | Sex*Minichromosome | F(1,12)=2.748 p=0.123 |
| 6E | 6 | 1% Triton X100 sol. amyloid-β_42_ | Sex | F(1,11)=6.212 p=0.03 |
| 6E | 6 | 1% Triton X100 sol. amyloid-β_42_ | Sex*Minichromosome | F(1,11)=1.946 p=0.191 |
| 6E | 12 | 1% Triton X100 sol. amyloid-β_42_ | Sex | F(1,12)=2.264 p=0.16 |
| 6E | 12 | 1% Triton X100 sol. amyloid-β_42_ | Sex*Minichromosome | F(1,12)=1.389 p=0.261 |
| 6F | 3 | 5M GndHcl soluble amyloid_42_ | Sex | F(1,12)=0.734 p=0.408 |
| 6F | 3 | 5M GndHcl sol. amyloid-β_42_ | Sex*Minichromosome | F(1,12)=0.983 p=0.341 |
| 6F | 6 | 5M GndHcl sol. amyloid-β_42_ | Sex | F(1,11)=1.451 p=0.254 |
| 6F | 6 | 5M GndHcl sol. amyloid-β_42_ | Sex*Minichromosome | F(1,11)=1.191 p=0.298 |
| 6F | 12 | 5M GndHcl sol. amyloid-β_42_ | Sex | F(1,12)=1.82 p=0.202 |
| 6F | 12 | 5M GndHcl sol. amyloid-β_42_ | Sex*Minichromosome | F(1,12)=0.696 p=0.421 |
|  |  |  |  |  |
| 6G | 3 | Tris sol. amyloid-β_40_ | Sex | F(1,12)=1.532 p=0.239 |
| 6G | 3 | Tris sol. amyloid-β_40_ | Sex*Duplication | F(1,12)=0.486 p=0.499 |
| 6G | 6 | Tris sol. amyloid-β_40_ | Sex | F(1,14)=0.631 p=0.44 |
| 6G | 6 | Tris sol. amyloid-β_40_ | Sex*Duplication | F(1,14)=0.026 p=0.875 |
| 6G | 12 | Tris sol. amyloid-β_40_ | Sex | F(1,15)=1.652 p=0.218 |
| 6G | 12 | Tris sol. amyloid-β_40_ | Sex*Duplication | F(1,15)=0.607 p=0.448 |
| 6H | 3 | 1% Triton X100 sol. amyloid-β_40_ | Sex | F(1,12)=0.406 p=0.536 |
| 6H | 3 | 1% Triton X100 sol. amyloid-β_40_ | Sex*Duplication | F(1,12)=0.004 p=0.949 |
| 6H | 6 | 1% Triton X100 sol. amyloid-β_40_ | Sex | F(1,14)=1.36 p=0.263 |
| 6H | 6 | 1% Triton X100 sol. amyloid-β_40_ | Sex*Duplication | F(1,14)=1.68 p=0.216 |
| 6H | 12 | 1% Triton X100 sol. amyloid-β_40_ | Sex | F(1,15)=0.298 p=0.593 |
| 6H | 12 | 1% Triton X100 sol. amyloid-β_40_ | Sex*Duplication | F(1,15)=0.66 p=0.429 |
| 6I | 3 | 5M GndHcl sol. amyloid-β_40_ | Sex | F(1,12)=0.136 p=0.719 |
| 6I | 3 | 5M GndHcl sol. amyloid-β_40_ | Sex*Duplication | F(1,12)=0.305 p=0.591 |
| 6I | 6 | 5M GndHcl sol. amyloid-β_40_ | Sex | F(1,14)=0.003 p=0.956 |
| 6I | 6 | 5M GndHcl sol. amyloid-β_40_ | Sex*Duplication | F(1,14)=0.098 p=0.758 |
| 6I | 12 | 5M GndHcl sol. amyloid-β_40_ | Sex | F(1,15)=2.111 p=0.167 |
| 6I | 12 | 5M GndHcl sol. amyloid-β_40_ | Sex*Duplication | F(1,15)=0.777 p=0.392 |
| 6J | 3 | Tris sol. amyloid-β_42_ | Sex | F(1,12)=1.328 p=0.272 |
| 6J | 3 | Tris sol. amyloid-β_42_ | Sex*Duplication | F(1,12)=0.387 p=.546x |
| 6J | 6 | Tris sol. amyloid-β_42_ | Sex | F(1,14)=0.679 p=0.424 |
| 6J | 6 | Tris sol. amyloid-β_42_ | Sex*Duplication | F(1,14)=0.957 p=0.345 |
| 6J | 12 | Tris sol. amyloid-β_42_ | Sex | F(1,14)=0.349 p=0.564 |
| 6J | 12 | Tris sol. amyloid-β_42_ | Sex*Duplication | F(1,14)=0.22 p=0.646 |
| 6K | 3 | 1% Triton X100 sol. amyloid-β_42_ | Sex | F(1,12)=0.003 p=0.960 |
| 6K | 3 | 1% Triton X100 sol. amyloid-β_42_ | Sex*Duplication | F(1,12)=0.164 p=0.693 |
| 6K | 6 | 1% Triton X100 sol. amyloid-β_42_ | Sex | F(1,14)=4.555 p=0.051 |
| 6K | 6 | 1% Triton X100 sol. amyloid-β_42_ | Sex*Duplication | F(1,14)=0.07 p=0.795 |
| 6K | 12 | 1% Triton X100 sol. amyloid-β_42_ | Sex | F(1,15)=0.405 p=0.534 |
| 6K | 12 | 1% Triton X100 sol. amyloid-β_42_ | Sex*Duplication | F(1,15)=0.64 p=0.436 |
| 6L | 3 | 5M GndHcl soluble amyloid_42_ | Sex | F(1,12)=0.377 p=0.551 |
| 6L | 3 | 5M GndHcl sol. amyloid-β_42_ | Sex*Duplication | F(1,12)=0.821 p=0.383 |
| 6L | 6 | 5M GndHcl sol. amyloid-β_42_ | Sex | F(1,14)=0.033 p=0.858 |
| 6L | 6 | 5M GndHcl sol. amyloid-β_42_ | Sex*Duplication | F(1,14)=1.079 p=0.316 |
| 6L | 12 | 5M GndHcl sol. amyloid-β_42_ | Sex | F(1,15)=0.475 p=0.501 |
| 6L | 12 | 5M GndHcl sol. amyloid-β_42_ | Sex*Duplication | F(1,15)=3.674 p=0.075 |
