# Supplementary material for: Age‐related behavioral and molecular landmarks in new mouse models for studying Alzheimer's disease in Down syndrome
Source: Alzheimers Dement. 2026 May 21;22(5):e71498. doi: 10.1002/alz.71498 (PMC13240120; doi:10.1002/alz.71498)
Supplement: Supplementary file 7 — Supporting Information: alz71498‐sup‐0007‐TableS5.docx [file ALZ-22-e71498-s004.docx]

Supplementary table 6

ANOVA summary statistics for the effect of sex and the interaction of sex with other variables in hippocampal samples, genotype effects presented in Supplementary Figures 5 and 6 as indicated.

| **Figure** | **Age (months)** | **Analyte** | **Variable/Interaction** | **ANOVA and Bonferroni**  **Post hoc p<0.05** |
| --- | --- | --- | --- | --- |
| S5A | 3 | Hippocampal FL-APP | Sex | F(1,24)=4.198 p=0.052 |
| S5A | 3 | Hippocampal FL-APP | Sex*Humanisation | F(1,24)=0.839 p=0.369 |
| S5A | 3 | Hippocampal FL-APP | Sex*Minichromosome | F(1,24)=0.923 p=0.346 |
| S5A | 3 | Hippocampal FL-APP | Sex*Humanisation*Minichromosome | F(1,24)=0.746 p=0.396 |
| S5A | 6 | Hippocampal FL-APP | Sex | F(1,23)=0.054 p=0.819 |
| S5A | 6 | Hippocampal FL-APP | Sex*Humanisation | F(1,23)=0.703 p=0.41 |
| S5A | 6 | Hippocampal FL-APP | Sex*Minichromosome | F(1,23)=0.466 p=0.502 |
| S5A | 6 | Hippocampal FL-APP | Sex*Humanisation*Minichromosome | F(1,23)=0.693 p=0.414 |
| S5A | 12 | Hippocampal FL-APP | Sex | F(1,24)=0.213 p=0.648 |
| S5A | 12 | Hippocampal FL-APP | Sex*Humanisation | F(1,24)=0.618 p=0.439 |
| S5A | 12 | Hippocampal FL-APP | Sex*Minichromosome | F(1,24)=3.773 p=0.064 |
| S5A | 12 | Hippocampal FL-APP | Sex*Humanisation*Minichromosome | F(1,24)=0.361 p=0.553 |
| S5B | 3 | Hippocampal CTF-α | Sex | F(1,24)=0.175 p=0.68 |
| S5B | 3 | Hippocampal CTF-α | Sex*Humanisation | F(1,24)=0.275 p=0.605 |
| S5B | 3 | Hippocampal CTF-α | Sex*Minichromosome | F(1,24)=0.051 p=0.824 |
| S5B | 3 | Hippocampal CTF-α | Sex*Humanisation*Minichromosome | F(1,24)=0.299 p=0.589 |
| S5B | 6 | Hippocampal CTF-α | Sex | F(1,23)=3.957 p=0.059 |
| S5B | 6 | Hippocampal CTF-α | Sex*Humanisation | F(1,23)=2.299 p=0.143 |
| S5B | 6 | Hippocampal CTF-α | Sex*Minichromosome | F(1,23)=0.062 p=0.806 |
| S5B | 6 | Hippocampal CTF-α | Sex*Humanisation*Minichromosome | F(1,23)=0.44 p=0.836 |
| S5B | 12 | Hippocampal CTF-α | Sex | F(1,24)=0.206 p=0.654 |
| S5B | 12 | Hippocampal CTF-α | Sex*Humanisation | F(1,24)=3.357 p=0.079 |
| S5B | 12 | Hippocampal CTF-α | Sex*Minichromosome | F(1,24)=2.261 p=0.146 |
| S5B | 12 | Hippocampal CTF-α | Sex*Humanisation*Minichromosome | F(1,24)=0.007 p=0.932 |
| S5C | 3 | Hippocampal CTF-β | Sex | F(1,24)=0.731 p=0.401 |
| S5C | 3 | Hippocampal CTF-β | Sex*Humanisation | F(1,24)=0.229 p=0.637 |
| S5C | 3 | Hippocampal CTF-β | Sex*Minichromosome | F(1,24)=0.107 p=0.746 |
| S5C | 3 | Hippocampal CTF-β | Sex*Humanisation*Minichromosome | F(1,24)=0.388 p=0.539 |
| S5C | 6 | Hippocampal CTF-β | Sex | F(1,23)=1.718 p=0.203 |
| S5C | 6 | Hippocampal CTF-β | Sex*Humanisation | F(1,23)=1.547 p=0.226 |
| S5C | 6 | Hippocampal CTF-β | Sex*Minichromosome | F(1,23)=1.346 p=0.258 |
| S5C | 6 | Hippocampal CTF-β | Sex*Humanisation*Minichromosome | F(1,23)=0.368 p=0.55 |
| S5C | 12 | Hippocampal CTF-β | Sex | F(1,24)=0.007 p=0.934 |
| S5C | 12 | Hippocampal CTF-β | Sex*Humanisation | F(1,24)=0.505 p=0.484 |
| S5C | 12 | Hippocampal CTF-β | Sex*Minichromosome | F(1,24)=0.005 p=0.943 |
| S5C | 12 | Hippocampal CTF-β | Sex*Humanisation*Minichromosome | F(1,24)=0.242 p=0.627 |
| S5D | 3 | Hippocampal CTF-β/CTF-α | Sex | F(1,24)=0.415 p=0.525 |
| S5D | 3 | Hippocampal CTF-β/CTF-α | Sex*Humanisation | F(1,24)=0.131 p=0.721 |
| S5D | 3 | Hippocampal CTF-β/CTF-α | Sex*Minichromosome | F(1,24)=0.361 p=0.554 |
| S5D | 3 | Hippocampal CTF-β/CTF-α | Sex*Humanisation*Minichromosome | F(1,24)=0.004 p=0.95 |
| S5D | 6 | Hippocampal CTF-β/CTF-α | Sex | F(1,23)=0.109 p=0.745 |
| S5D | 6 | Hippocampal CTF-β/CTF-α | Sex*Humanisation | F(1,23)=0.182 p=0.673 |
| S5D | 6 | Hippocampal CTF-β/CTF-α | Sex*Minichromosome | F(1,23)=2.531 p=0.125 |
| S5D | 6 | Hippocampal CTF-β/CTF-α | Sex*Humanisation*Minichromosome | F(1,23)=0.644 p=0.43 |
| S5D | 12 | Hippocampal CTF-β/CTF-α | Sex | F(1,24)=0.049 p=0.826 |
| S5D | 12 | Hippocampal CTF-β/CTF-α | Sex*Humanisation | F(1,24)=0.014 p=0.906 |
| S5D | 12 | Hippocampal CTF-β/CTF-α | Sex*Minichromosome | F(1,24)=0.437 p=0.515 |
| S5D | 12 | Hippocampal CTF-β/CTF-α | Sex*Humanisation*Minichromosome | F(1,24)=0.822 p=0.373 |
|  |  |  |  |  |
| S6A | 3 | Hippocampal FL-APP | Sex | F(1,24)=0.093 p=0.763 |
| S6A | 3 | Hippocampal FL-APP | Sex*Humanisation | F(1,24)=0.029 p=0.867 |
| S6A | 3 | Hippocampal FL-APP | Sex*Duplication | F(1,24)=0.075 p=0.786 |
| S6A | 3 | Hippocampal FL-APP | Sex*Humanisation*Duplication | F(1,24)=0.015 p=0.905 |
| S6A | 6 | Hippocampal FL-APP | Sex | F(1,27)=0.683 p=0.416 |
| S6A | 6 | Hippocampal FL-APP | Sex*Humanisation | F(1,27)=2.999 p=0.095 |
| S6A | 6 | Hippocampal FL-APP | Sex*Duplication | F(1,27)=0.489 p=0.49 |
| S6A | 6 | Hippocampal FL-APP | Sex*Humanisation*Duplication | F(1,27)=4.161 p=0.051 |
| S6A | 12 | Hippocampal FL-APP | Sex | F(1,26)=0.369 p=0.549 |
| S6A | 12 | Hippocampal FL-APP | Sex*Humanisation | F(1,26)=0.716 p=0.405 |
| S6A | 12 | Hippocampal FL-APP | Sex*Duplication | F(1,26)=0.01 p=0.92 |
| S6A | 12 | Hippocampal FL-APP | Sex*Humanisation*Duplication | F(1,26)=0.147 p=0.705 |
| S6A | 3 | Hippocampal CTF-α | Sex | F(1,24)=0.055 p=0.816 |
| S6B | 3 | Hippocampal CTF-α | Sex*Humanisation | F(1,24)<0.001 p=0.989 |
| S6B | 3 | Hippocampal CTF-α | Sex*Duplication | F(1,24)=0.005 p=0.943 |
| S6B | 3 | Hippocampal CTF-α | Sex*Humanisation*Duplication | F(1,24)=0.158 p=0.695 |
| S6B | 6 | Hippocampal CTF-α | Sex | F(1,27)=0.145 p=0.707 |
| S6B | 6 | Hippocampal CTF-α | Sex*Humanisation | F(1,27)=0.026 p=0.873 |
| S6B | 6 | Hippocampal CTF-α | Sex*Duplication | F(1,27)=0.367 p=0.55 |
| S6B | 6 | Hippocampal CTF-α | Sex*Humanisation*Duplication | F(1,27)=0.601 p=0.445 |
| S6B | 12 | Hippocampal CTF-α | Sex | F(1,26)=0.001 p=0.974 |
| S6B | 12 | Hippocampal CTF-α | Sex*Humanisation | F(1,26)=0.399 p=0.533 |
| S6B | 12 | Hippocampal CTF-α | Sex*Duplication | F(1,26)=0.366 p=0.551 |
| S6B | 12 | Hippocampal CTF-α | Sex*Humanisation*Duplication | F(1,26)=0.004 p=0.947 |
| S6C | 3 | Hippocampal CTF-β | Sex | F(1,24)=0.372 p=0.547 |
| S6C | 3 | Hippocampal CTF-β | Sex*Humanisation | F(1,24)=0.156 p=0.696 |
| S6C | 3 | Hippocampal CTF-β | Sex*Duplication | F(1,24)=0.192 p=0.665 |
| S6C | 3 | Hippocampal CTF-β | Sex*Humanisation*Duplication | F(1,24)=0.033 p=0.857 |
| S6C | 6 | Hippocampal CTF-β | Sex | F(1,27)=0.186 p=0.67 |
| S6C | 6 | Hippocampal CTF-β | Sex*Humanisation | F(1,27)=0.074 p=0.787 |
| S6C | 6 | Hippocampal CTF-β | Sex*Duplication | F(1,27)=0.007 p=0.935 |
| S6C | 6 | Hippocampal CTF-β | Sex*Humanisation*Duplication | F(1,27)=1.029 p=0.319 |
| S6C | 12 | Hippocampal CTF-β | Sex | F(1,26)=0.002 p=0.961 |
| S6C | 12 | Hippocampal CTF-β | Sex*Humanisation | F(1,26)=0.121 p=0.731 |
| S6C | 12 | Hippocampal CTF-β | Sex*Duplication | F(1,26)=0.331 p=0.57 |
| S6C | 12 | Hippocampal CTF-β | Sex*Humanisation*Duplication | F(1,26)=0.013 p=0.91 |
| S6C | 3 | Hippocampal CTF-β/CTF-α | Sex | F(1,24)=0.36 p=0.554 |
| S6D | 3 | Hippocampal CTF-β/CTF-α | Sex*Humanisation | F(1,24)=0.251 p=0.621 |
| S6D | 3 | Hippocampal CTF-β/CTF-α | Sex*Duplication | F(1,24)=0.144 p=0.708 |
| S6D | 3 | Hippocampal CTF-β/CTF-α | Sex*Humanisation*Duplication | F(1,24)=0.001 p=0.974 |
| S6D | 6 | Hippocampal CTF-β/CTF-α | Sex | F(1,27)=1.17 p=0.289 |
| S6D | 6 | Hippocampal CTF-β/CTF-α | Sex*Humanisation | F(1,27)=0.183 p=0.672 |
| S6D | 6 | Hippocampal CTF-β/CTF-α | Sex*Duplication | F(1,27)=0.281 p=0.601 |
| S6D | 6 | Hippocampal CTF-β/CTF-α | Sex*Humanisation*Duplication | F(1,27)=0.73 p=0.401 |
| S6D | 12 | Hippocampal CTF-β/CTF-α | Sex | F(1,26)=0.116 p=0.737 |
| S6D | 12 | Hippocampal CTF-β/CTF-α | Sex*Humanisation | F(1,26)=0.219 p=0.644 |
| S6D | 12 | Hippocampal CTF-β/CTF-α | Sex*Duplication | F(1,26)=0.001 p=0.976 |
| S6D | 12 | Hippocampal CTF-β/CTF-α | Sex*Humanisation*Duplication | F(1,26)=0.155 p=0.697 |
