# Supplementary material for: Age‐related behavioral and molecular landmarks in new mouse models for studying Alzheimer's disease in Down syndrome
Source: Alzheimers Dement. 2026 May 21;22(5):e71498. doi: 10.1002/alz.71498 (PMC13240120; doi:10.1002/alz.71498)
Supplement: Supplementary file 8 — Supporting Information: alz71498‐sup‐0008‐TableS6.docx [file ALZ-22-e71498-s005.docx]

**Supplementary table 4: Summary of the main behavioural phenotypes observed in the DS models compared to wild-type control**

| **Genotype / Age** | | ***App^H/H^*** | | **Ts66Yah** | | **Ts68Yah** | |
| --- | --- | --- | --- | --- | --- | --- | --- |
| **Test /** Variable | | **3M** | **9M** | **3M** | **9M** | **3M** | **9M** |
| **Circadian activity** | Locomotor activity | = | = | + | + | + | + |
|  | Rears | = | = | + | + | + | +hr |
| **Open Field 1** | Locomotor activity | + | + | = | + | + | + |
| **Elevated plus maze** | Total arm entries | = | = | = | + | + | + |
|  | Open arm entries | + | + | = | = | + | + |
|  | % time open arm | = | + | = | = | + | + |
| **Y maze** | Spontaneous alternation | = | = | = | - | = | = |
|  | Activity (visits) | = | = | = | = | + | + |
| **Novel Object**  **Recognition** | Recognition index 1h of retention | = | - | - | - | - | - |
|  | Recognition index 24h of retention | - | - | - | - | - | - |
| **Morris water**  **maze** | Training phase | = | = | = | = | = | = |
|  | Probe test | = | = | - | - | = | = |
